# Investigation of Genetic Variation Underlying Central Obesity amongst South Asians

**DOI:** 10.1371/journal.pone.0155478

**Published:** 2016-05-19

**Authors:** William R. Scott, Weihua Zhang, Marie Loh, Sian-Tsung Tan, Benjamin Lehne, Uzma Afzal, Juan Peralta, Richa Saxena, Sarju Ralhan, Gurpreet S. Wander, Kiymet Bozaoglu, Dharambir K. Sanghera, Paul Elliott, James Scott, John C. Chambers, Jaspal S. Kooner

**Affiliations:** 1 Epidemiology and Biostatistics, Imperial College London, Norfolk Place, London, United Kingdom; 2 National Heart and Lung Institute, Imperial College London, Hammersmith Hospital, Du Cane Road, London, United Kingdom; 3 Ealing Hospital NHS Trust, Southall, Middlesex, United Kingdom; 4 Genomics Computer Centre, South Texas Diabetes and Obesity Institute, University of Texas at the Rio Grande Valley, Brownsville, Texas, United States of America; 5 Broad Institute of Massachusetts Institute of Technology and Harvard, Massachusetts General Hospital, Cambridge, MA, United States of America; 6 Hero DMC Heart Institute, Ludhiana, Punjab, India; 7 Genomics and Systems Biology, Baker IDI Heart and Diabetes Institute, Melbourne, VIC Australia; 8 Department of Pediatrics, Section of Genetics, College of Medicine, University of Oklahoma Health Sciences Center, Oklahoma City, OK, United States of America; 9 Department of Pharmaceutical Sciences, College of Pharmacy, University of Oklahoma Health Sciences Center, Oklahoma City, OK, United States of America; 10 Oklahoma Center for Neuroscience, University of Oklahoma Health Sciences Center, Oklahoma City, OK, United States of America; 11 MRC-PHE Centre for Environment and Health, Imperial College London, Norfolk Place, London, United Kingdom; 12 Imperial College Healthcare NHS Trust, Du Cane Road, London, United Kingdom; Wake Forest School of Medicine, UNITED STATES

## Abstract

South Asians are 1/4 of the world’s population and have increased susceptibility to central obesity and related cardiometabolic disease. Knowledge of genetic variants affecting risk of central obesity is largely based on genome-wide association studies of common SNPs in Europeans. To evaluate the contribution of DNA sequence variation to the higher levels of central obesity (defined as waist hip ratio adjusted for body mass index, WHR) among South Asians compared to Europeans we carried out: i) a genome-wide association analysis of >6M genetic variants in 10,318 South Asians with focused analysis of population-specific SNPs; ii) an exome-wide association analysis of ~250K SNPs in protein-coding regions in 2,637 South Asians; iii) a comparison of risk allele frequencies and effect sizes of 48 known WHR SNPs in 12,240 South Asians compared to Europeans. In genome-wide analyses, we found no novel associations between common genetic variants and WHR in South Asians at P<5x10^-8^; variants showing equivocal association with WHR (P<1x10^-5^) did not replicate at P<0.05 in an independent cohort of South Asians (N = 1,922) or in published, predominantly European meta-analysis data. In the targeted analyses of 122,391 population-specific SNPs we also found no associations with WHR in South Asians at P<0.05 after multiple testing correction. Exome-wide analyses showed no new associations between genetic variants and WHR in South Asians, either individually at P<1.5x10^-6^ or grouped by gene locus at P<2.5x10^−6^. At known WHR loci, risk allele frequencies were not higher in South Asians compared to Europeans (P = 0.77), while effect sizes were unexpectedly smaller in South Asians than Europeans (P<5.0x10^-8^). Our findings argue against an important contribution for population-specific or cosmopolitan genetic variants underlying the increased risk of central obesity in South Asians compared to Europeans.

## Introduction

Central obesity is a major risk factor for insulin resistance, type-2 diabetes (T2D), and cardiovascular disease (CVD) [1,2,3,4]. South Asians, who comprise 1/4 of the world’s population, are at high risk of developing central obesity compared to Europeans [5,6,7,8,9], and this is widely considered to underlie their 2–4 fold excess risk of T2D and CVD [10,11,12,13,14]. Central obesity is heritable amongst South Asians [15,16], consistent with an important genetic contribution. Genome-wide association studies (GWAS) predominantly carried out in people of European ancestry have identified genetic loci underlying central obesity [17,18,19,20,21,22]. Few studies have investigated genetic factors underlying central obesity amongst South Asians [23,24].

To investigate whether genetic variation accounts for the increased risk of central obesity amongst South Asians compared to Europeans, we carried out South Asian-specific genome- and exome-wide association analyses and related our results to published European findings. We selected waist hip ratio adjusted for body mass index (WHR) as a measure of central obesity. We focused on population-specific SNPs present amongst South Asians but not Europeans to test whether these influence WHR amongst South Asians. We also targeted cosmopolitan SNPs associated with WHR in previous GWAS, and SNPs in protein coding regions which are considered to have a higher probability of functional relevance.

## Materials and Methods

### Design

**Fig 1:**
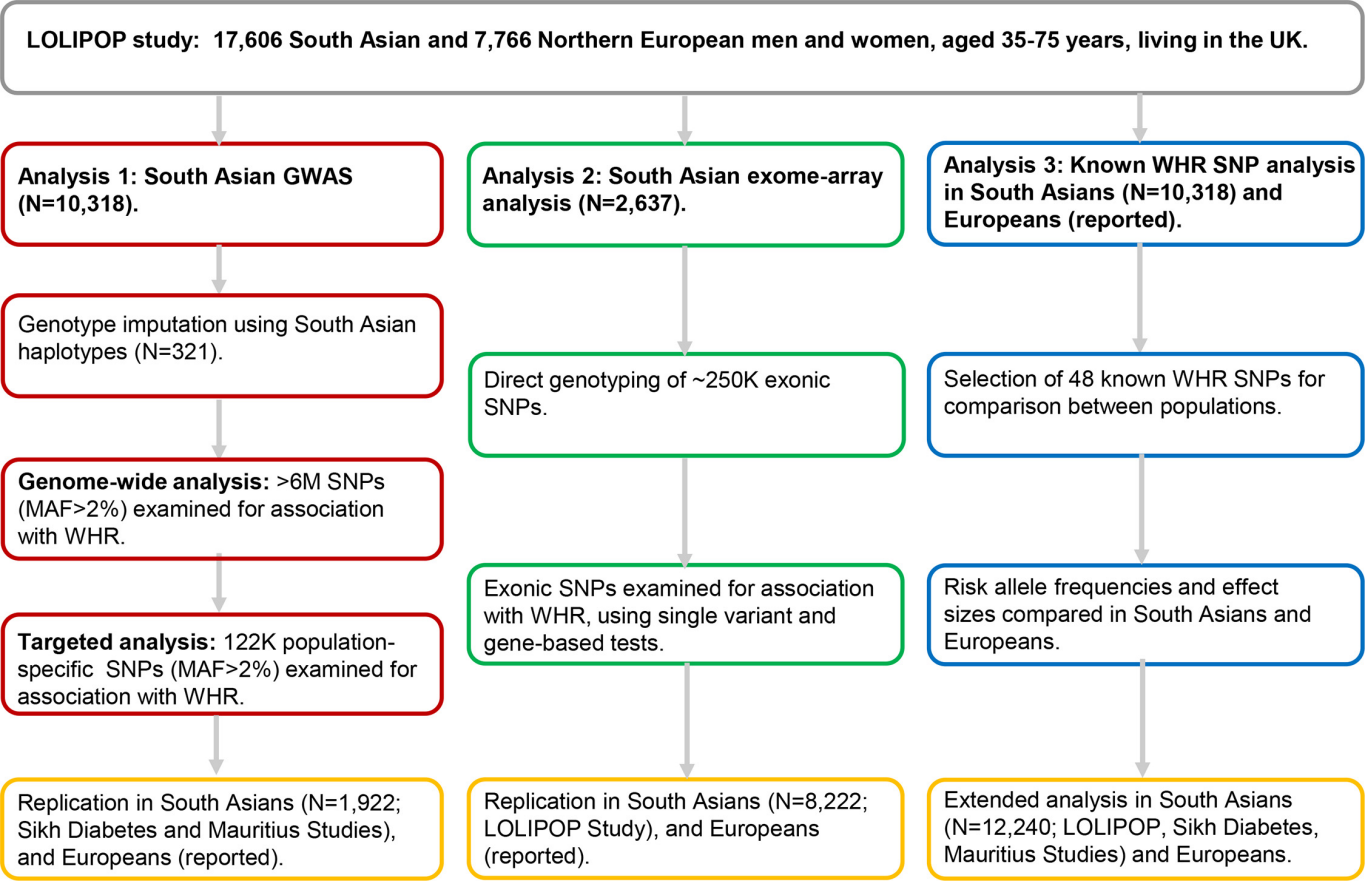
Study Design.

We investigated the contribution of population-specific and cosmopolitan DNA sequence variation to the increased risk of central obesity in South Asians using genome- and exome-wide association analyses. The study design is described in Fig 1.

### Population and phenotype

Samples for the discovery analyses were selected from the London Life Sciences Population (LOLIPOP) study, an ongoing population-based cohort of 17,606 South Asian and 7,766 Northern European men and women, aged 35–75 years, recruited from the lists of 58 general practitioners in West London. South Asians were recruited to the study if all 4 grandparents were born in the Indian Subcontinent (countries of India, Pakistan, Sri Lanka or Bangladesh). Data on medical history, current prescribed medication, and cardiovascular risk factors were obtained by a trained research nurse using an interviewer-administered questionnaire. Country of birth of participants, parents, and grandparents were recorded together with language and religion, for assignment of ethnic subgroups. Physical measurements included blood pressure (mean of 3 readings, taken with an Omron 705CP), height, weight, waist and hip circumference. Blood was collected after an 8 hour fast for plasma glucose, total and HDL cholesterol, triglycerides, insulin and high sensitivity C-reactive protein. The research was approved by the West London Research Ethics Committee (reference number: 07/H0712/150), and all participants gave written informed consent. Replication testing was done amongst 1,922 South Asian participants from the Sikh Diabetes and Mauritius Family Studies (S1 Appendix; S1 Table) [25,26,27,28].

Waist hip ratio adjusted for body mass index (WHR) was used as a measure of central obesity. This allows assessment of abdominal fat independent of differences in overall adiposity [19].

### Genotyping

#### Sample selection

We selected 10,318 South Asians for genome-wide association, and 2,637 South Asians for exome-wide association (2,096 individuals were present in both groups). We also carried out genome-wide association in 2,148 Europeans to enable comparison between ethnic groups. Sample selection criteria and characteristics of participants are summarised in S1 Table.

#### Genome-wide association

Genotyping platforms, calling algorithms, and quality control measures used for genome-wide association are summarised in S2 Table. Hidden relatedness or duplicate samples were sought using identity-by-descent methods implemented in PLINK; samples with evidence for relatedness (pi_hat ≥ 0.5) were identified and one sample was retained. Principal components analysis was carried out in Eigensoft v3.0; eigenvalues inconsistent with either South Asian or European ancestry were removed.

Imputation of unmeasured genotypes was carried out amongst South Asians using sequencing data from 321 South Asian participants in the LOLIPOP study [29], enabling the investigation of population-specific genetic variants. In Europeans, imputation of unmeasured genotypes was carried out using the cosmopolitan 1000 genomes reference panel (phase 1, version 3). All imputation was performed using IMPUTE2; markers with low imputation info score (<0.4), Hardy-Weinberg equilibrium P<1.0x10^−6^, or minor allele frequency (MAF) <2% were excluded. We assessed the accuracy of our South Asian-specific imputation by comparing imputed genotypes with corresponding direct genotyping results from our exome-wide dataset among overlapping individuals (6,480 variants with MAF >2%). Compared to the exome-wide dataset, mean sensitivity and specificity of the imputed SNPs were 99.41% (0.03) and 99.40% (0.03) respectively. Mean concordance between direct and imputed genotypes was 98.31% (0.04). 6,571,328 SNPs were generated for South Asian-specific genome-wide association analysis.

#### Exome-wide association

We genotyped 247,870 variants in protein-coding regions among 2,637 South Asians, using the Illumina HumanExome Beadchip and OmniExpressExome (S2 Table). Variants were called using Gencall, followed by post-processing with zCall to improve calling of rare variants. Samples with a SNP call rate <95% were removed, as were SNPs with call rate <97%, MAF <1%, or Hardy-Weinberg equilibrium P<1.0x10^−6^. 245,496 SNPs passed quality control, of which 35,368 SNPs had MAF >1%.

### Statistical analyses

#### South Asian-specific GWAS

Single variants were examined for association with WHR using linear regression using SNPTEST; covariate adjustments were made for age, sex, BMI, and 10 principal components to control for residual population stratification. SNP-trait associations were examined under additive, dominant and recessive inheritance models. Results were normalised using an inverse normal transformed ranked scale, to enable comparison with reported SNPs shown to be associated with WHR in previous GWAS. Each genotyping platform was analysed separately, and results were combined by fixed effects inverse variance meta-analysis within METAL. Heterogeneity was evaluated using the Cochran's Q statistic. A significance threshold of P<5x10^-8^ was used for the discovery GWAS.

We refined our approach by focusing on a smaller number of genetic variants from our genome-wide analysis which were population-specific (present in South Asians but not present in Europeans within the 1000 genomes reference panel phase 1, version 3). Amongst the >6 million SNPs investigated in our genome-wide analysis we identified 122,391 South Asian-specific SNPs with MAF >2% which we examined for association with WHR. For the analyses of population-specific genetic variants, statistical significance was inferred at P<0.05 after Bonferroni correction for the number of SNPs tested, thus enhancing study power compared to genome-wide association.

#### South Asian-specific exome-wide association

Exonic variants were examined for association with WHR using linear regression and an additive genetic model within RAREMETALWORKER, with adjustments for age, sex, and 10 principal components of ancestry. Results for each genotyping platform were analysed separately and combined by meta-analysis within METAL. Common, low-frequency, and rare variants were also grouped by genetic locus for gene-based association analysis within RAREMETAL (CMC, Madsen-Browning, Variable Threshold, and SKAT methods). We selected significance thresholds of P<1.5x10^-6^ and P<2.5x10^−6^ for the single variant and gene-based analyses respectively (Bonferroni corrected).

#### Replication analysis

We carried out replication testing of all genetic variants showing suggestive association with WHR amongst South Asians in genome-wide (P<1x10^-5^) and exome-wide (P<1x10^-3^) analyses. Genome-wide results were tested for replication in an independent cohort of South Asians (N = 1,922; S1 and S2 Tables; S1 Appendix) [25,26,27], and in published, predominantly European meta-analysis data from the Giant Consortium (2012–2014 release) [20]. For our exome-wide results we performed replication testing among independent samples from our discovery South Asian GWAS analysis (N = 8,222). A replication significance threshold of P<0.05 was selected.

#### Population comparisons

We carried out a literature search to identify previously reported SNPs associated with central obesity in GWAS at P<5x10^-8^. We found 38 SNPs associated with WHR in men and women [19,22], and 11 SNPs associated in women alone [20,22].

We selected 48 of the 49 known WHR SNPs for comparison between South Asians and Europeans. One SNP, rs7801581 in *HOXA11*, was not available within our South Asian sample. We analysed the 48 known WHR SNPs for association with WHR in South Asians at P<0.05. We examined for systematic differences in risk allele frequencies and in effect sizes between South Asian participants in LOLIPOP and reported European results using the sign test; assessing whether the observed South Asian results, categorised individually as higher or lower than the European reference, differed significantly from the binomial probability distribution (expected value of 0.5). The contribution of known genetic variants to the excess in WHR among South Asians compared to Europeans in the LOLIPOP study was quantified by multivariate linear regression. To extend our analysis, we examined the 48 WHR SNPs in an independent cohort of South Asians (N = 1,922), and used meta-analysis to combine the results with our findings among South Asian participants in LOLIPOP.

### Study power

Our South Asian GWAS has 80% power to identify single variants explaining 0.40% of the variation in WHR among 10,318 South Asians at P<5x10^-8^ (S3A Table); power to detect variants with effects equivalent to the average effect size of reported WHR variants is <1% at genome-wide significance. In our exome-array analysis we have 80% power to detect variants explaining 1.0% of the variation in WHR among 2,637 South Asians at P<1.5x10^-6^ (S3A Table). For our population comparison of previously reported WHR SNPs from GWAS, we estimate between 10 and 84% power to replicate individual variants at P<0.05 among 10,318 South Asians (S3B Table; or 15 and 91% among 12,240 South Asians, combined discovery and replication cohorts).

**Fig 2:**
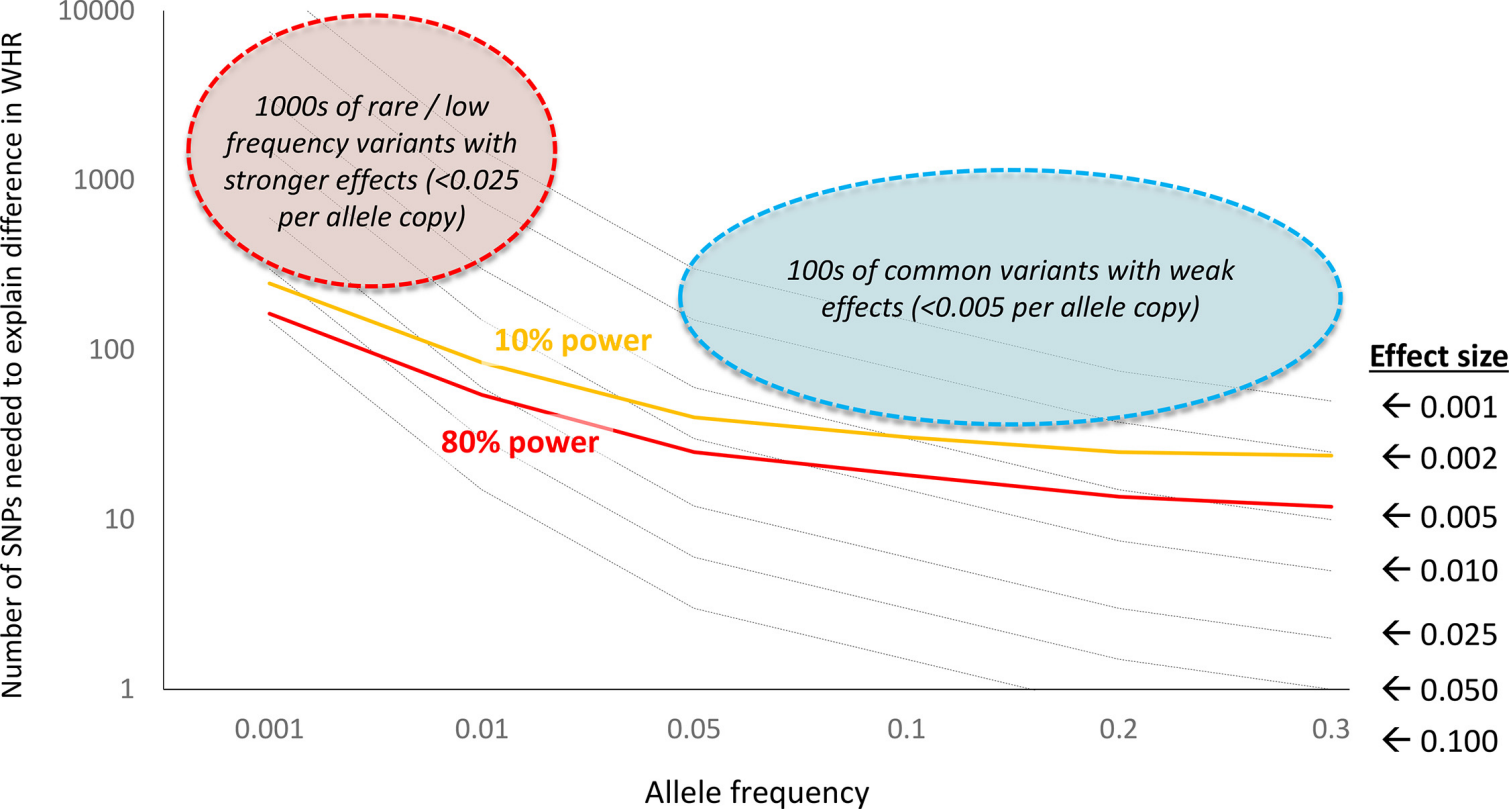
Contribution of population-specific genetic variants to increased WHR amongst South Asians compared to Europeans. Results are shown as the number of SNPs needed to fully explain the difference in WHR between the populations across a range of effect sizes (per allele copy, dashed black lines). Superimposed are lines showing the power (10% power, orange line; 80% power, red line) of the current study to detect common and low frequency genetic variants associated with WHR amongst South Asians. Results show that our study sample size is sufficient to identify common variants with effect size >0.005 per allele copy, and rare / infrequent variants with effect size >0.025 (untransformed β values). At these effect sizes, there would need to be 10s to 100s of population-specific genetic variants to explain increased WHR amongst South Asians. At effect sizes smaller than those identifiable in the current study, there would need to be 100s of common variants or 1000s of rare / low frequency variants that are population-specific and associated with WHR, to account for increased WHR amongst South Asians.

To enhance study power we carried out targeted analyses of South Asian-specific SNPs. We simulated the numbers of population-specific SNPs that would be required to explain the increase in WHR amongst South Asians compared to Europeans as a function of allele frequency and effect size (Fig 2). Amongst 10,318 South Asians we have >80% power to detect common, population-specific variants associated with WHR with an effect size of >0.005 per allele copy (untransformed β value). At this effect size there would need to be in excess of >20 common, population-specific common genetic variants associated with WHR amongst South Asians (Fig 2).

## Results

### Population characteristics

The clinical characteristics of the 25,372 South Asian and European participants of the LOLIPOP Study are shown in Table 1. Compared to Europeans, South Asians have higher waist hip ratio (0.94 (0.08) v. 0.91 (0.08); P<0.001) despite similar body mass index (BMI) (27.4 (4.6) v. 27.5 (5.2); P = 0.23). South Asians also have a higher prevalence of T2D and CVD, higher fasting glucose, insulin, HOMA-IR, HbA1c, and triglycerides, with lower HDL-cholesterol compared to Europeans. Waist hip ratio remained higher amongst South Asians compared to Europeans after adjustment for differences in age, gender, and BMI (Table 2).

**Table 1:** Characteristics of participants in the LOLIPOP Study.

	South Asians	Europeans	P value
*n*	*17*,*606*	*7*,*766*	
Age (yrs)	50.5 (11.2)	52.3 (11.6)	8.3E-29
Male (%)	61	60	1.7E-02
Coronary heart disease (%)	11	6	1.1E-32
Type-2 diabetes (%)	19	7	9.4E-106
Hypertension (%)	29	20	1.1E-47
Treated type-2 diabetes (%)	13	4	3.2E-90
Treated hypertension (%)	28	19	5.6E-45
Treated dyslipidaemia (%)	20	13	6.5E-34
Body mass index (kg/m2)	27.4 (4.6)	27.5 (5.2)	2.3E-01
**Waist hip ratio**	**0.94 (0.08)**	**0.91 (0.08)**	**3.9E-154**
Systolic BP (mmHg)	131 (19)	131 (19)	6.2E-01
Diastolic BP (mmHg)	80 (11)	79 (11)	2.5E-12
Cholesterol (mmol/L)	5.2 (1.1)	5.4 (1.1)	4.9E-45
LDL cholesterol (mmol/L)	3.2 (0.9)	3.3 (0.9)	4.3E-28
HDL cholesterol (mmol/L)	1.3 (0.3)	1.4 (0.4)	4.0E-186
Triglycerides (mmol/L)	1.7 (1.2)	1.5 (1.1)	7.7E-32
Glucose (mmol/L)	5.8 (2.0)	5.4 (1.5)	3.8E-65
Insulin (mU/L)	13.1 (12.2)	9.9 (10.1)	7.6E-85
HbA1c	6.0 (1.3)	5.5 (0.9)	3.0E-289
HOMA-IR	3.6 (4.3)	2.6 (3.7)	1.0E-63

Results are presented as mean (standard deviation), or % for categorical variables; comparisons using student t- and chi-squared tests.

**Table 2:** Multivariate regression analysis of waist hip ratio in South Asian compared to European participants in the LOLIPOP Study.

Adjustments made	WHR difference (95% CI) South Asians vs. Europeans	P value
Age, Sex	0.030 (0.027–0.034)	6.3E-68
Age, Sex + BMI	0.038 (0.035–0.041)	2.3E-132
Age, Sex + BMI + 37 WHR SNPs	0.038 (0.034–0.042)	6.0E-76
Age, Sex + BMI + 37 WHR SNPs + 11 additional WHR SNPs	0.037 (0.033–0.042)	3.1E-61

37 WHR SNPs–previously reported associations with WHR in men and women (P<5x10^-8^); 11 additional WHR SNPs–previously reported associations with WHR in women alone (P<5x10^-8^).

### South Asian-specific GWAS

#### Genome-wide analysis

There were no genetic variants (MAF >2%) associated with WHR at genome-wide significance under additive, dominant, or recessive inheritance models (S4 Table and S1 Fig). Comparisons of the distributions for observed and expected association results revealed little evidence for test statistic inflation (Lambdas additive = 1.025, dominant = 1.015, recessive = 1.015, S2 Fig). Variants showing equivocal association with WHR under an additive model (5x10^-8^<P<1x10^-5^) did not replicate at P<0.05, either in an independent cohort of South Asians (N = 1,922) or in published, predominantly European data for the Giant Consortium (S4 Table).

#### Population-specific SNPs

We examined population-specific genetic variants for association with WHR in South Asians, adopting 4 MAF thresholds: i. >2% (N = 122,391); ii. >5% (N = 38,639); iii. >10% (N = 7,349); and iv. >20% (N = 596). None of these population-specific genetic variants were found to be associated with WHR in South Asians at P<0.05 after Bonferroni correction for the number of SNPs tested in their respective allele frequency window (S5 Table). In addition, the observed distributions of association statistics did not deviate from the expected null distributions arguing against enrichment for association with WHR amongst the population-specific SNPs tested (Lambda = 1.022, S3 Fig).

### South Asian-specific exome-wide association

None of the examined variants (MAF >1%) were found to be associated with WHR in South Asians at P<1.5x10^-6^ (S6 Table and S4 Fig), and we found no evidence of deviation of the observed distribution of the test statistics from that expected under the null hypothesis (Lambda = 0.996, S5 Fig). In replication testing of equivocal variants (1.5x10^-6^<P<1x10^-3^), only the rs17778003 SNP in *ZFAT* showed evidence of association with WHR at P<0.05, in South Asians (P = 7.2x10^-3^) but not in Europeans (P = 0.58) (S6 Table). We found no genes associated with WHR at Bonferroni genome-wide significance threshold of P<2.5x10^−6^ using gene-based association analyses (S7 Table).

### Comparison of known genetic variants

We examined 37 known WHR SNPs for association with variation in WHR amongst South Asian men and women participating in the LOLIPOP study. Only 4 achieved nominal significance (rs6556301 near *FGFR4*, P = 6.3x10^-03^; rs984222 in *TBX15*, P = 6.6x10^-3^; rs1011731 in *DNM3*, P = 1.8x10^-2^; rs12608504 near *JUND*, P = 2.7x10^-02^; Table 3). For the 11 SNPs known to be associated with WHR in women alone, 2 had a nominally significant effect in South Asian women (rs4684854 near *PPARG*, P = 1.3x10^-2^; rs1534696 in *SNX10*, P = 2.7x10^-3^; Table 3).

**Table 3:** Comparison of 48 known WHR SNPs in South Asians and Europeans.

Marker Name	Nearest Gene	E/A	Ref	EAF	β (SEM)	P value	*n*	Dir	Power	EAF	β (SEM)	P value	*n*
				**SOUTH ASIANS COMBINED LOLIPOP**						**EUROPEANS COMBINED REPORTED**			
**rs9491696**	*RSPO3*	G/C	(19)	0.49	-0.011 (0.012)	3.5E-01	10,318	-	84%	0.48	0.042	1.8E-40	113,582
**rs6905288**	*VEGFA*	A/G	(19)	0.81	0.014 (0.018)	4.2E-01	10,318	+	71%	0.56	0.036	5.9E-25	95,430
**rs984222**	*TBX15-WARS2*	G/C	(19)	0.48	0.032 (0.012)	**6.6E-03**	10,318	+	64%	0.64	0.034	8.7E-25	109,623
**rs1055144**	*NFE2L3*	T/C	(19)	0.39	0.018 (0.012)	1.3E-01	10,318	+	63%	0.21	0.04	1.0E-24	113,636
**rs10195252**	*GRB14*	T/C	(19)	0.72	-0.005 (0.013)	7.0E-01	10,318	-	63%	0.6	0.033	2.1E-24	102,449
**rs4846567**	*LYPLAL1*	G/T	(19)	0.76	0.008 (0.014)	5.6E-01	10,318	+	57%	0.72	0.034	6.9E-21	91,820
**rs1011731**	*DNM3-PIGC*	G/A	(19)	0.44	0.028 (0.012)	**1.8E-02**	10,318	+	50%	0.43	0.028	9.5E-18	92,018
**rs718314**	*ITPR2-SSPN*	G/A	(19)	0.24	0.003 (0.014)	8.1E-01	10,318	+	46%	0.26	0.03	1.1E-17	107,503
**rs1294421**	*LY86*	G/T	(19)	0.45	-0.007 (0.039)	8.5E-01	10,318	-	49%	0.61	0.028	1.8E-17	102,189
**rs1443512**	*HOXC13*	A/C	(19)	0.32	0.002 (0.013)	9.0E-01	10,318	+	47%	0.24	0.031	6.4E-17	112,353
**rs4823006**	*ZNRF3-KREMEN1*	A/G	(19)	0.44	-0.009 (0.012)	4.8E-01	10,318	-	36%	0.57	0.023	1.1E-11	93,911
**rs6784615**	*NISCH-STAB1*	T/C	(19)	0.96	-0.037 (0.03)	2.1E-01	10,318	-	30%	0.94	0.043	3.8E-10	109,028
**rs6861681**	*CPEB4*	A/G	(19)	0.13	0.007 (0.017)	6.7E-01	10,318	+	31%	0.34	0.022	1.9E-09	85,722
**rs6795735**	*ADAMTS9*	C/T	(19)	0.28	-0.002 (0.014)	8.6E-01	10,318	-	41%	0.59	0.025	1.1E+13	84,480
**rs4765219**	*CCDC92*	C/A	(22)	0.79	0.005 (0.015)	7.2E-01	10,005	+	47%	0.67	0.028 (0.004)	1.6E-15	209,807
**rs979012**	*BMP2*	T/C	(22)	0.44	0.017 (0.012)	1.7E-01	10,005	+	45%	0.34	0.027 (0.004)	3.3E-14	209,941
**rs17451107**	*LEKR1*	T/C	(22)	0.73	0.010 (0.014)	4.9E-01	9,504	+	45%	0.61	0.026 (0.004)	1.1E-12	207,795
**rs4081724**	*CEBPA*	G/A	(22)	0.96	0.031 (0.031)	3.1E-01	9,505	+	43%	0.85	0.035 (0.005)	7.4E-12	207,418
**rs4646404**	*PEMT*	G/A	(22)	0.87	-0.031 (0.019)	1.0E-01	9505	-	45%	0.67	0.027 (0.004)	1.4E-11	198,196
**rs12679556**	*MSC*	G/T	(22)	0.44	0.021 (0.012)	9.0E-02	10,005	+	39%	0.25	0.027 (0.004)	2.1E-11	203,826
**rs7759742**	*BTNL2*	A/T	(22)	0.58	-0.005 (0.013)	7.0E-01	9,504	-	38%	0.51	0.023 (0.003)	4.4E-11	208,263
**rs6090583**	*EYA2*	A/G	(22)	0.54	-0.015 (0.013)	2.5E-01	9,505	-	35%	0.48	0.022 (0.003)	6.2E-11	209,435
**rs1440372**	*SMAD6*	C/T	(22)	0.74	0.009 (0.014)	5.3E-01	10,005	+	35%	0.71	0.024 (0.004)	1.1E-10	207,447
**rs7801581**	*HOXA11*	T/C	(22)	NA	NA	NA	NA	NA	NA	0.24	0.027 (0.004)	3.7E-10	195,215
**rs1569135**	*CALCRL*	A/G	(22)	0.43	0.010 (0.012)	4.0E-01	10,005	+	32%	0.53	0.021 (0.003)	5.6E-10	209,906
**rs905938**	*DCST2*	T/C	(22)	0.85	0.003 (0.017)	8.6E-01	9,505	+	35%	0.74	0.025 (0.004)	7.3E-10	207,867
**rs12608504**	*JUND*	A/G	(22)	0.33	0.028 (0.013)	**2.7E-02**	10,005	+	33%	0.36	0.022 (0.004)	8.8E-10	209,990
**rs8042543**	*KLF13*	C/T	(22)	0.79	0.017 (0.016)	2.7E-01	9,505	+	34%	0.78	0.026 (0.004)	1.2E-09	208,255
**rs1385167**	*MEIS1*	G/A	(22)	0.18	0.019 (0.016)	2.2E-01	10,005	+	31%	0.15	0.029 (0.005)	1.9E-09	206,619
**rs10919388**	*GORAB*	C/A	(22)	0.75	0.003 (0.014)	8.3E-01	9,505	+	34%	0.72	0.024 (0.004)	3.2E-09	181,049
**rs10804591**	*PLXND1*	A/C	(22)	0.65	0.005 (0.013)	6.7E-01	10,005	+	30%	0.80	0.025 (0.004)	6.6E-09	209,921
**rs8030605**	*RFX7*	A/G	(22)	0.26	-0.007 (0.014)	6.0E-01	10,005	-	32%	0.14	0.030 (0.005)	8.8E-09	208,374
**rs10991437**	*ABCA1*	A/C	(22)	0.09	0.003 (0.021)	8.9E-01	10,005	+	28%	0.11	0.031 (0.005)	1.0E-08	209,941
**rs224333**	*GDF5*	G/A	(22)	0.44	0.012 (0.013)	3.2E-01	10,004	+	29%	0.62	0.020 (0.004)	2.6E-08	208,025
**rs6556301**	*FGFR4*	T/G	(22)	0.41	0.034 (0.013)	**6.3E-03**	9,505	+	33%	0.36	0.022 (0.004)	2.6E-08	178,874
**rs303084**	*SPATA5-FGF2*	A/G	(22)	0.73	0.019 (0.014)	1.6E-01	10,005	+	26%	0.80	0.023 (0.004)	3.9E-08	209,941
**rs9991328**	*FAM13A*	T/C	(22)	0.58	0.002 (0.012)	8.7E-01	10,005	+	28%	0.49	0.019 (0.003)	4.5E-08	209,925
**rs11231693**	*MACROD1-VEGFB*	A/G	(22)	0.03	0.041 (0.042)	3.2E-01	9,505	+	29%	0.06	0.041 (0.008)	4.5E-08	198,072
								**27 / 37**					
				**SOUTH ASIANS WOMEN LOLIPOP**						**EUROPEANS WOMEN REPORTED**			
**rs4684854**	*PPARG*	C/G	(20)	0.61	0.088 (0.035)	**1.3E-02**	1,463	+	17%	0.43	0.037	4.2E-14	96,472
**rs10478424**	*HSD17B4*	A/T	(20)	0.73	-0.026 (0.039)	5.1E-01	1,463	-	14%	0.78	0.039	3.5E-09	73,066
**rs7830933**	*NKX2-6*	A/G	(22)	0.73	0.073 (0.041)	7.3E-02	1,443	+	13%	0.77	0.037 (0.005)	1.2E-12	116,567
**rs9687846**	*MAP3K1*	A/G	(22)	0.17	-0.018 (0.047)	7.1E-01	1,443	-	14%	0.19	0.041 (0.006)	3.8E-12	115,897
**rs2925979**	*CMIP*	T/C	(22)	0.30	0.051 (0.038)	1.7E-01	1,443	+	12%	0.31	0.032 (0.005)	3.4E-11	115,431
**rs12454712**	*BCL2*	T/C	(22)	0.45	0.043 (0.034)	2.1E-01	1,443	+	15%	0.61	0.035 (0.006)	1.1E-09	96,182
**rs8066985**	*KCNJ2*	A/G	(22)	0.43	0.006 (0.034)	8.7E-01	1,443	+	10%	0.50	0.026 (0.005)	4.0E-09	116,683
**rs7917772**	*SFXN2*	A/G	(22)	0.40	0.031 (0.036)	3.8E-01	1,443	+	11%	0.62	0.027 (0.005)	5.5E-09	116,514
**rs1776897**	*HMGA1*	G/T	(22)	0.05	0.055 (0.077)	4.7E-01	1,443	+	12%	0.08	0.052 (0.009)	6.8E-09	100,516
**rs3805389**	*NMU*	A/G	(22)	0.26	-0.019 (0.039)	6.3E-01	1,443	-	10%	0.28	0.027 (0.005)	4.6E-08	116,226
**rs1534696**	*SNX10*	C/A	(22)	0.42	-0.087 (0.039)	**2.7E-02**	1,443	-	11%	0.44	0.027 (0.005)	5.4E-08	111,643
								**7 / 11**					

Abbreviations: E/A–effect and alternative alleles; EAF—effect allele frequencies; β (SEM)—β coefficients (standard error of mean) per change of WHR-increasing allele on WHR (adjusted for BMI, inverse normal transformed ranked scale); P value—for association with WHR; Dir—direction of effect allele compared to reported European results.

**Fig 3:**
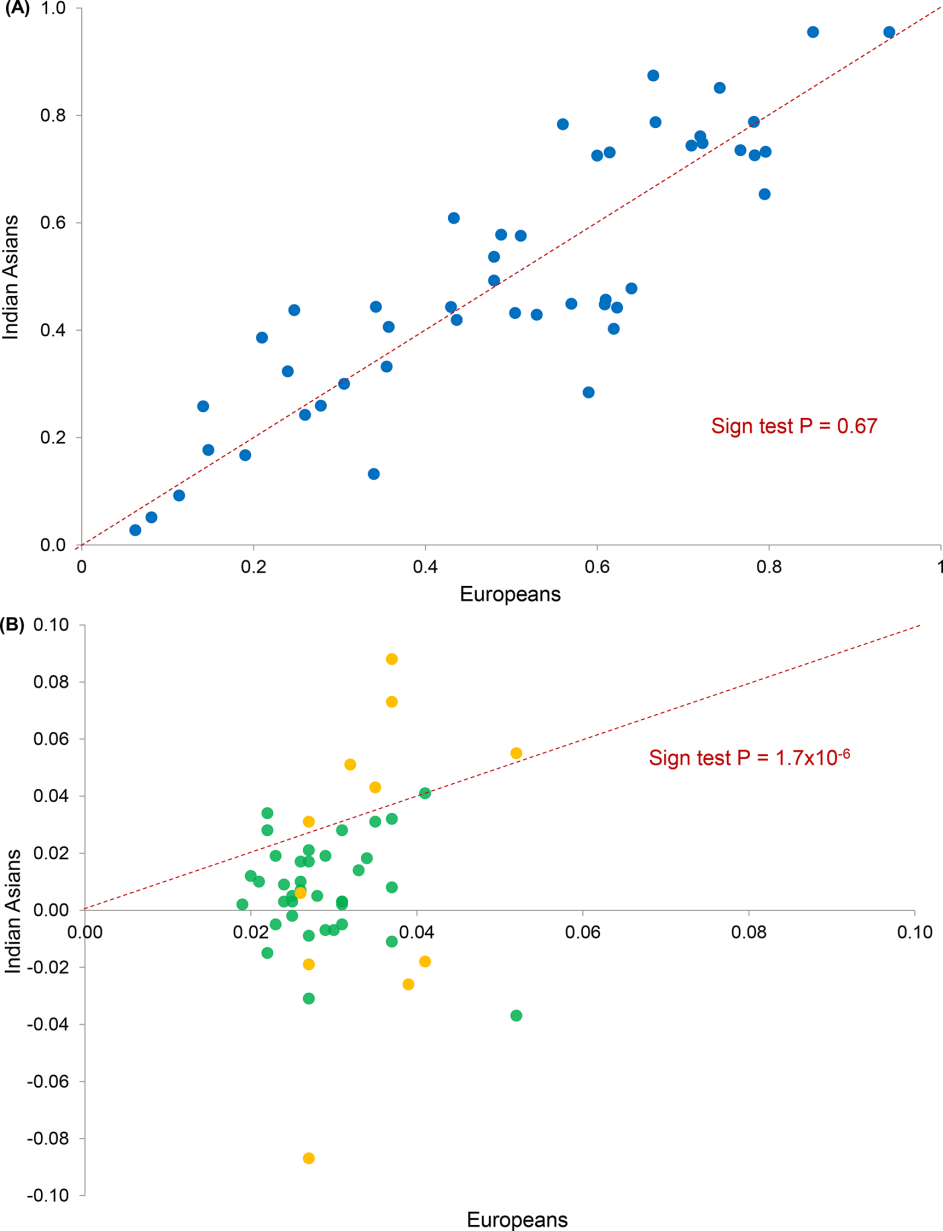
Known WHR SNPs. **(A) Comparison of risk allele frequencies in South Asians (N = 10,318) and Europeans (reported). (B) Comparison of effect sizes (β WHR) in South Asians and Europeans.** Green–men and women combined (37 SNPs in South Asians (N = 10,318) and Europeans (reported)); orange–women alone (11 SNPs in South Asians (N = 1,463) and Europeans (reported)).

We compared overall effect allele frequencies and effect sizes for these 48 SNPs between South Asian participants in the LOLIPOP Study and reported European results. Effect allele frequencies were similar between the two groups (sign test P = 0.67, Fig 3A). Effect sizes for WHR were consistently lower in South Asians compared to Europeans (sign test P<1.7x10^-6^, Fig 3B). Inclusion of results from a further 1,922 independent South Asians did not increase the number of SNPs achieving nominal significance (S8 Table), and did not alter comparisons of effect allele frequencies or effect sizes between populations (sign test P = 0.77 and P<5.0x10^-8^ respectively, S6A and S6B Fig).

Together the 48 established WHR SNPs explained 0.7% of variation in WHR, equivalent to 2.5% of the reported ~27% heritability [16], among South Asians (LOLIPOP Study participants, men and women combined). When included in multivariate regression analyses examining the differences in WHR between the two populations, these variants did not account for any of the excess risk of central obesity observed in South Asians compared to Europeans (Table 2).

## Discussion and Conclusion

Central obesity, characterised by accumulation of excess body fat in an abdominal distribution, is a leading risk factor for T2D, CVD, and premature mortality [1,2,3,4,30,31]. Central obesity is more prevalent amongst South Asians compared to Europeans [5,6,7,8,9], but the mechanisms underlying this are not well understood. Family studies show that central obesity is heritable in South Asians [15,16]. Current knowledge of genetic loci influencing central obesity risk is however largely based on the study of common variants using GWAS among Europeans [17,18,19,20,21,22].

We carried out a GWAS of WHR in South Asians, using imputation of population-specific and cosmopolitan genetic variants identified through next-generation sequencing in 321 South Asians [29]. We found that none of the >6 million evaluated variants were associated with variation in WHR among 10,318 South Asians at genome-wide significance. We then carried out a targeted analysis of population-specific SNPs to test whether these might account for increased WHR amongst South Asians. Despite focus on a smaller number of genetic variants, enhancing study power compared to genome-wide association, we again found no genetic variants associated with WHR in South Asians. In parallel we carried out an exome-array analysis among 2,637 South Asians to provide more comprehensive characterisation of genetic variation in protein coding regions, which are considered to have a higher probability of functional relevance. We found a single variant, rs17778003 in *ZFAT*, showing weak association with WHR in South Asians, but no novel associations were identified at P<1.5x10^-6^. Further we found no evidence of genes showing enrichment for common, low-frequency or rare protein-coding variants underlying WHR in South Asians.

We modelled the number of population-specific SNPs required to explain the increase in WHR amongst South Asians compared to Europeans as a function of allele frequency and effect size, as well as study power to detect these variations. Our simulations show we are well powered to identify variants with modest phenotypic effects in our sample of 10,318 South Asians (Fig 2). At these effect sizes there would need to be upwards of 20 population-specific genetic variants associated with WHR amongst South Asians. In contrast we find none. We therefore conclude that a small number of common, population-specific genetic variants with modest effect size do not account for increased WHR amongst South Asians. Indeed, if increased WHR amongst South Asians is the result population-specific genetic variants, this is a polygenic disorder comprising very many genetic variants (>20) that are uncommon (MAF <2%) and / or have small effect size (<0.005 increase in WHR per allele copy).

Differences in risk allele frequencies and effect sizes at known adiposity loci, including *FTO* and *MC4R*, have been observed between South Asians and Europeans [23,32]. We compared reported SNPs at 48 established WHR loci in South Asians and in Europeans to investigate whether these variants underlie the excess risk of central obesity observed in South Asians. When compared with published European results, we observed limited evidence for replication at known WHR SNPs among South Asians; only 34 of 48 WHR loci showed directionally consistent effects on WHR in South Asians. When all 48 known WHR variants were examined together we observed no systematic differences in risk allele frequencies, and consistently smaller effect sizes, in South Asians compared to Europeans. Combined these variants accounted for <1% of phenotypic variation in WHR among South Asians, and did not contribute to the excess of WHR observed in South Asians compared to Europeans. The reasons for the lack of replication of known WHR SNPs in South Asians are not known but may include: (i) genetic loci with European-specific effects; (ii) stronger relationships between tag and causal SNPs in Europeans because of differences in haplotype structure; (iii) inflated effect sizes in the European discovery sample due to winner's curse; (iv) greater phenotypic heterogeneity in South Asians, who have smaller stature but greater central adiposity than Europeans, with reduced power to detect SNP-trait associations. Nevertheless, our findings robustly demonstrate that known WHR SNPs do not account for the increased risk of central obesity amongst South Asians compared to Europeans.

Mechanisms underlying central obesity risk among South Asians remain unclear. We have not excluded the possibility of a genetic contribution through multiple common variants with small effects, rare variants with larger phenotypic effects, or structural variations that are inadequately captured using current analytic platforms. Environmental influences have an important role in central adiposity [1,33,34,35,36,37,38], and may contribute to increased risk of central obesity in South Asians. For example differences in lifestyle, such as lower levels of physical activity [39,40,41,42], and higher levels of total calorie, refined starch and saturated fat intake [43,44,45,46], have been reported in South Asians compared to Europeans. Similarly, the prevalence of low birth weight, which is associated with accelerated childhood weight gain [47,48,49] and future development of central obesity and metabolic disturbance [50,51,52,53,54,55], is higher in South Asians than Europeans [56,57,58,59,60,61]. Another explanation is that population-specific epigenomic modifications [62,63,64,65], which regulate gene expression and phenotypic variation without change in DNA sequence [66,67], contribute to central obesity predisposition amongst South Asians. These modifications in DNA methylation, histone modification, and chromatin remodelling, can be transmitted through the germline and modified by environmental exposures [66,68,69,70,71], providing a compelling putative mechanism for unexplained phenotypic variation. Future initiatives combining new, more targeted analysis strategies and larger sample sizes will be required to elucidate the relative roles of these genomic and environmental factors in the excess of central obesity among South Asians.

## Supporting Information

S1 Appendix South Asian replication cohort description. (DOCX)

S1 Fig South Asian GWAS–Association of SNPs with WHR by chromosome position. (A) Additive inheritance model. (B) Dominant inheritance model. (C) Recessive inheritance model.
(TIF)

S2 Fig South Asian GWAS–Relationship of observed association statistics with those expected under the null distribution. (A) Additive inheritance model (Lambda = 1.025). (B) Dominant inheritance model (Lambda = 1.015). (C) Recessive inheritance model (Lambda = 1.015).
(TIF)

S3 Fig South-Asian GWAS targeted analysis of population-specific SNPs. Relationship of observed association statistics with those expected under the null distribution (MAF>2%, Lambda = 1.022).
(TIF)

S4 Fig South Asian exome-array analysis–Association of SNPs with WHR by chromosome position. (TIF)

S5 Fig South Asian exome-array analysis–Relationship of observed association statistics with those expected under the null distribution (Lambda = 0.996). (TIF)

S6 Fig Known WHR SNPs. (A) Comparison of risk allele frequencies in South Asians, extended analysis (N = 12,240) and Europeans (reported). (B) Comparison of effect sizes (β WHR) in South Asians and Europeans, extended analysis. Green–men and women combined (37 SNPs in South Asians (N = 12,240) and Europeans (reported)); orange–women alone (11 SNPs in South Asians (N = 2,363) and Europeans (reported)).
(TIF)

S1 Table Characteristics of participants in the genotyping cohorts. (DOCX)

S2 Table Genotyping cohort platforms and quality metrics. (DOCX)

S3 Table Study power to identify single variants associated with variation in WHR amongst South Asians, and replicate known WHR SNPs amongst South Asians. (DOCX)

S4 Table South Asian GWAS–Top ranking single markers at P<1x10^-5^ under an additive, dominant and recessive inheritance models inheritance model. (DOCX)

S5 Table South Asian GWAS targeted analysis–Top ranking population-specific markers under an additive inheritance model. (DOCX)

S6 Table South Asian exome-array analysis–Top ranking single markers at P<1x10^-3^, with replication among South Asians (GWAS) and Europeans (GIANT consortium meta-analysis [20]). (DOCX)

S7 Table South Asian exome-array analysis–Top ranking gene-based association results at P<1x10^-3^. (DOCX)

S8 Table Comparison of 48 known WHR SNPs in South Asians (N = 12,240; extended analysis) and Europeans. (DOCX)
