# Supplementary material for: Investigation of Genetic Variation Underlying Central Obesity amongst South Asians
Source: PLoS One. 2016 May 19;11(5):e0155478. doi: 10.1371/journal.pone.0155478 (PMC4873263; doi:10.1371/journal.pone.0155478)
Supplement: S1 Appendix — (DOCX) [file pone.0155478.s001.docx]

**Appendix S1. South Asian replication cohort description.**

**Replication cohorts.**

*Sikh Diabetes Study (SDS).* Participants of SDS were recruited from Sikhs living in the Northern states of India, including Punjab, Haryana, Himachal Pradesh, Delhi, and Jammu and Kashmir ([1](#_ENREF_1)). Participants comprised individuals with type-2 diabetes mellitus (T2D; defined as physician diagnosis on treatment, a fasting plasma glucose level of ≥7.0mmol/L, or 2-hour post glucose load level of ≥11.1mmol/L, on more than one occasion with symptoms of diabetes) or controls with no prior history of diabetes and normal glucose tolerance (fasting glucose <6mmol/L, post glucose <7.8mmol/L). All participants provided a written informed consent for investigations. All SDS protocols and consent documents were reviewed and approved by the University of Oklahoma and the University of Pittsburgh Institutional Review Boards as well as the Human Subject Protection Committees at the participating hospitals and institutes in India.

*Mauritius Family Study.* Mauritius is a subtropical island located in the south western South Ocean with a population of about 1.2 million. An estimated 70% of the population are of South Asian origin (54% Hindu and 16% Muslim), 2% are of Chinese origin and 28% are of the “general‟ population, which mainly comprises people with mixed African and Malagasy ancestry with some European and South admixture (Creoles). A population based survey was undertaken in 1998 that included participants who were 20 years and older, with a total of 6,291 participants examined ([2](#_ENREF_2), [3](#_ENREF_3))). The Mauritian Family Study consisted of a cohort of 400 individuals in 20 large extended pedigrees. The participants ranged in age from 19 to 93 and included equal numbers of males and females. Approximately 22% had been diagnosed with type 2 diabetes. Participants of self-reported South Asian ancestry were included in the present study. All subjects not taking diabetes medication had a 2-h 75-g oral glucose (glucose monohydrate) tolerance test (OGTT). Venous blood samples were drawn at baseline fasting and at 2 hours post ingestion of glucose and were centrifuged and separated immediately. Plasma glucose was measured using the YSI glucose analyzer (Yellow Springs Instruments, OH, USA). Glucose tolerance status was determined according to 1999 WHO criteria. Diabetes was diagnosed if subjects reported a history of diabetes and were taking hypoglycaemic medication, or the fasting plasma glucose level was >=7.0 mmol/L and/or the 2-h value was >=11.1 mmol/L. Normal glucose tolerance was assigned if the fasting plasma glucose level was < 6.1 mmol/L and the 2-h value was < 6.1 mmol/L and the 2-h value was <7.8 mmol/L. The Mauritius Family Study was a collaborative effort between the International Diabetes Institute, the Mauritius Ministry of Health and Quality of Life and the University of Mauritius SSR Centre for Medical Studies and Research ([4](#_ENREF_4)).

**Replication testing.**

*Phenotype.* Waist hip ratio adjusted for body mass index (WHR) was used as a measure of central obesity in both replication cohorts. WHR was transformed to enable comparison of replication with discovery results (inverse normal transformed ranked scale).

*Sikh Diabetes Study.* 729 participants without T2D and 799 with T2D were genotyped using Illumina’s 660 Quad array, and genotypes were called using BeadStudio. Samples with <95% call rate were excluded, as were SNPs with call rate <95% or that deviated from Hardy-Weinberg equilibrium at P<1.0x10−6. Principal components analysis was carried out in Eigensoft v3.0, and eigenvalues inconsistent with either South Asian ancestry were removed. Imputation of unmeasured genotypes was carried out in Impute 2, using the 1000 genomes multi-ethnic reference panel and South Asian panel (LOLIPOP). Markers with low imputation info score (<0.4) or Hardy-Weinberg equilibrium P<1.0x10−6 were excluded. Single variants were examined for association with WHR using linear regression and an additive genetic model within SNPTEST; covariate adjustments were made for age, sex, and 10 principal components to control for residual population stratification. Results for participants with T2D and without T2D were analysed separately, and combined with results across all replication studies.

*Mauritius Family Study.* Genome-wide association scans were carried out in 20 families comprising 394 individuals. Their genomes where sequenced with an Illumina HiSeq2000 sequencer. Raw sequencing data from FASTQ files was aligned to the UCSC's Human Genome Reference release 19 (hg19) using BWA ([5](#_ENREF_5)), then recalibrated and realigned with GATK ([6](#_ENREF_6)). Genotypes were called using SAMtools ([7](#_ENREF_7)) and unmeasured or mistyped genotypes were imputed using ShapeIt2 ([8](#_ENREF_8)). An empirical kinship matrix was built from all of the directly genotyped markers within LDAK2. Associations of SNPs with WHR were tested, adjusting for age and sex, using the measured genotype approach (MGA) implemented in SOLAR ([9-11](#_ENREF_9)), which uses an additive genetic variance component model to account for the phenotypic correlation between family members.

*Meta-analysis*. A fixed effects meta-analysis was used to combine the results for each SNP across all replication studies and then in a combined analysis with results from the discovery genome-wide association analysis. Heterogeneity was evaluated using the Cochran's Q statistic. Statistical significance was inferred at *P*<0.05 in the replication stage. For the combined analysis of genome-wide and replication data, genome-wide significance was inferred at *P*<5×10^−8^.

**Acknowledgments.**

*Sikh Diabetes Study.* This work was supported by the National Institute of Health grant numbers KO1TW006087 funded by the Fogarty International Center; R01DK082766 funded by National Institute of Diabetes and Digestive and Kidney Diseases; and a seed grant from University of Oklahoma Health Sciences Center, Oklahoma City, USA.

*Mauritius Family Study*. The authors gratefully acknowledge the valuable contributions and support of the Mauritius Ministry of Health and Quality of Life and their health community workers, also Dr Navaratnam Kotea and staff at the SSR Centre, University of Mauritius. The authors further extend their gratitude to the individuals who participated in the study. This work was supported by the Australian Government National Health and Medical Research Council NHMRC project grant numbers 1020285 and 1037916, by the Victorian Government’s OIS Program, and partially funded by US National Institutes of Health Grant DK-25446. The SOLAR statistical genetics computer package is supported by a grant from the US National Institute of Mental Health (MH059490).

**References.**

**1. Sanghera DK, Bhatti JS, Bhatti GK, Ralhan SK, Wander GS, Singh JR, et al. The Khatri Sikh Diabetes Study (SDS): study design, methodology, sample collection, and initial results. Hum Biol. [Clinical Trial, Phase I Research Support, N.I.H., Extramural Research Support, Non-U.S. Gov't]. 2006 Feb;78(1):43-63.**

**2. Soderberg S, Zimmet P, Tuomilehto J, de Courten M, Dowse GK, Chitson P, et al. High incidence of type 2 diabetes and increasing conversion rates from impaired fasting glucose and impaired glucose tolerance to diabetes in Mauritius. J Intern Med. [Research Support, Non-U.S. Gov't Research Support, U.S. Gov't, P.H.S.]. 2004 Jul;256(1):37-47.**

**3. Soderberg S, Zimmet P, Tuomilehto J, de Courten M, Dowse GK, Chitson P, et al. Increasing prevalence of Type 2 diabetes mellitus in all ethnic groups in Mauritius. Diabetic medicine : a journal of the British Diabetic Association. [Research Support, Non-U.S. Gov't Research Support, U.S. Gov't, P.H.S.]. 2005 Jan;22(1):61-8.**

**4. Jowett JB, Diego VP, Kotea N, Kowlessur S, Chitson P, Dyer TD, et al. Genetic influences on type 2 diabetes and metabolic syndrome related quantitative traits in Mauritius. Twin Res Hum Genet. [Multicenter Study Research Support, N.I.H., Extramural Research Support, Non-U.S. Gov't]. 2009 Feb;12(1):44-52.**

**5. Li H, Durbin R. Fast and accurate short read alignment with Burrows-Wheeler transform. Bioinformatics. [Research Support, Non-U.S. Gov't]. 2009 Jul 15;25(14):1754-60.**

**6. McKenna A, Hanna M, Banks E, Sivachenko A, Cibulskis K, Kernytsky A, et al. The Genome Analysis Toolkit: a MapReduce framework for analyzing next-generation DNA sequencing data. Genome Res. [Research Support, N.I.H., Extramural]. 2010 Sep;20(9):1297-303.**

**7. Li H, Handsaker B, Wysoker A, Fennell T, Ruan J, Homer N, et al. The Sequence Alignment/Map format and SAMtools. Bioinformatics. [Research Support, N.I.H., Extramural Research Support, Non-U.S. Gov't]. 2009 Aug 15;25(16):2078-9.**

**8. O'Connell J, Gurdasani D, Delaneau O, Pirastu N, Ulivi S, Cocca M, et al. A general approach for haplotype phasing across the full spectrum of relatedness. PLoS genetics. [Research Support, Non-U.S. Gov't]. 2014 Apr;10(4):e1004234.**

**9. Aulchenko YS, de Koning DJ, Haley C. Genomewide rapid association using mixed model and regression: a fast and simple method for genomewide pedigree-based quantitative trait loci association analysis. Genetics. [Research Support, Non-U.S. Gov't]. 2007 Sep;177(1):577-85.**

**10. Boerwinkle E, Chakraborty R, Sing CF. The use of measured genotype information in the analysis of quantitative phenotypes in man. I. Models and analytical methods. Ann Hum Genet. [Research Support, U.S. Gov't, P.H.S.]. 1986 May;50(Pt 2):181-94.**

**11. Almasy L, Blangero J. Multipoint quantitative-trait linkage analysis in general pedigrees. Am J Hum Genet. [Research Support, U.S. Gov't, P.H.S.]. 1998 May;62(5):1198-211.**
