# Supplementary figures and images for: Investigation of Genetic Variation Underlying Central Obesity amongst South Asians
Source: PLoS One. 2016 May 19;11(5):e0155478. doi: 10.1371/journal.pone.0155478 (PMC4873263; doi:10.1371/journal.pone.0155478)

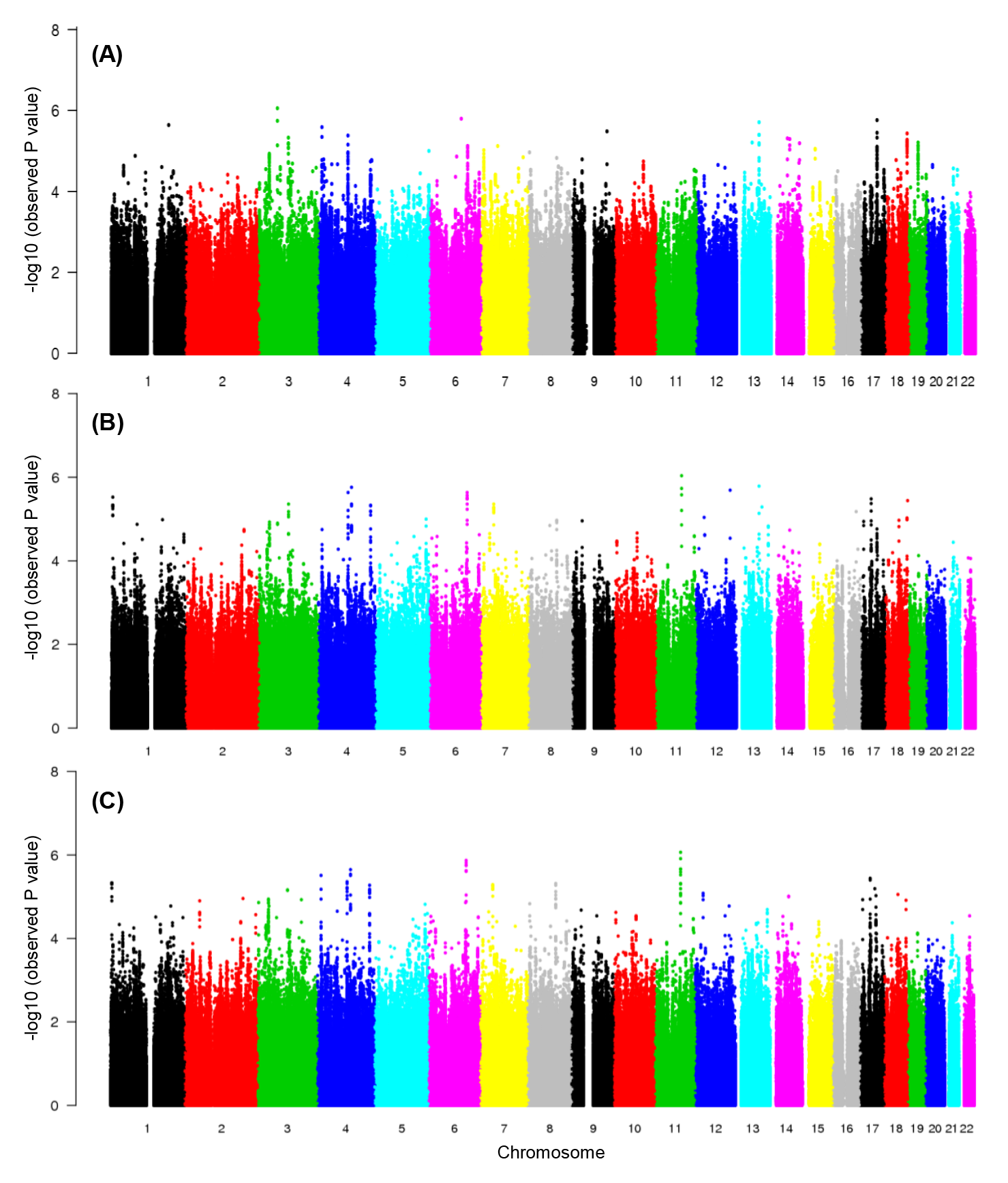

Supplement: S1 Fig — (A) Additive inheritance model. (B) Dominant inheritance model. (C) Recessive inheritance model. (TIF) [file pone.0155478.s002.tif]

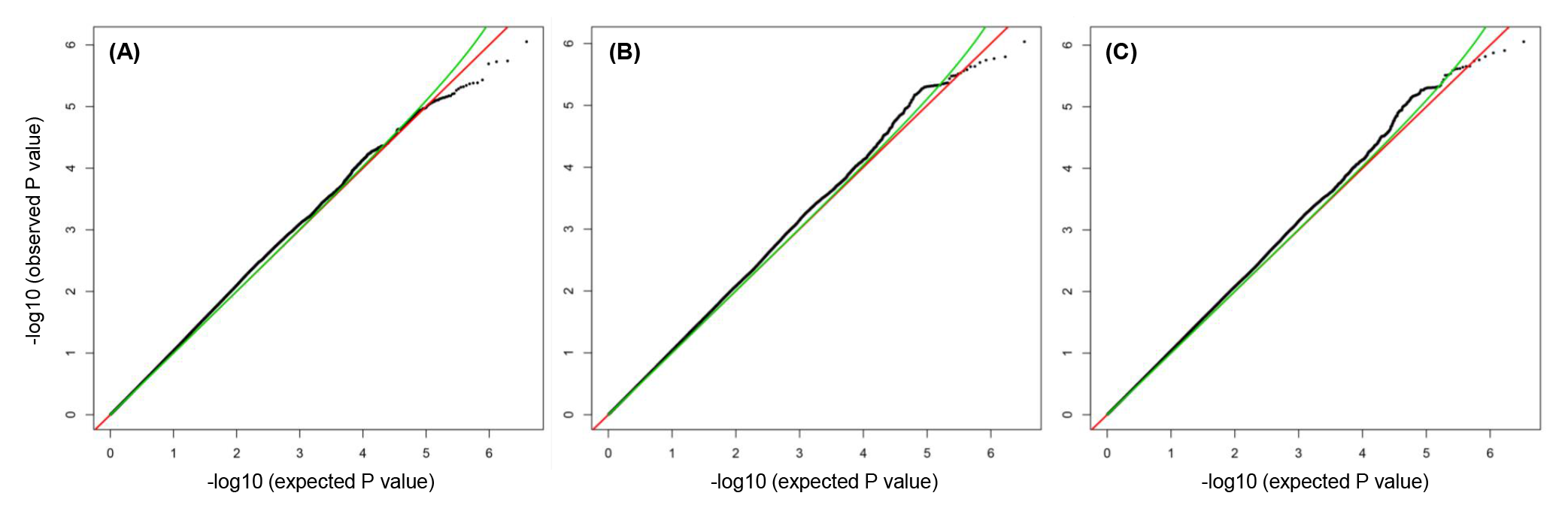

Supplement: S2 Fig — (A) Additive inheritance model (Lambda = 1.025). (B) Dominant inheritance model (Lambda = 1.015). (C) Recessive inheritance model (Lambda = 1.015). (TIF) [file pone.0155478.s003.tif]

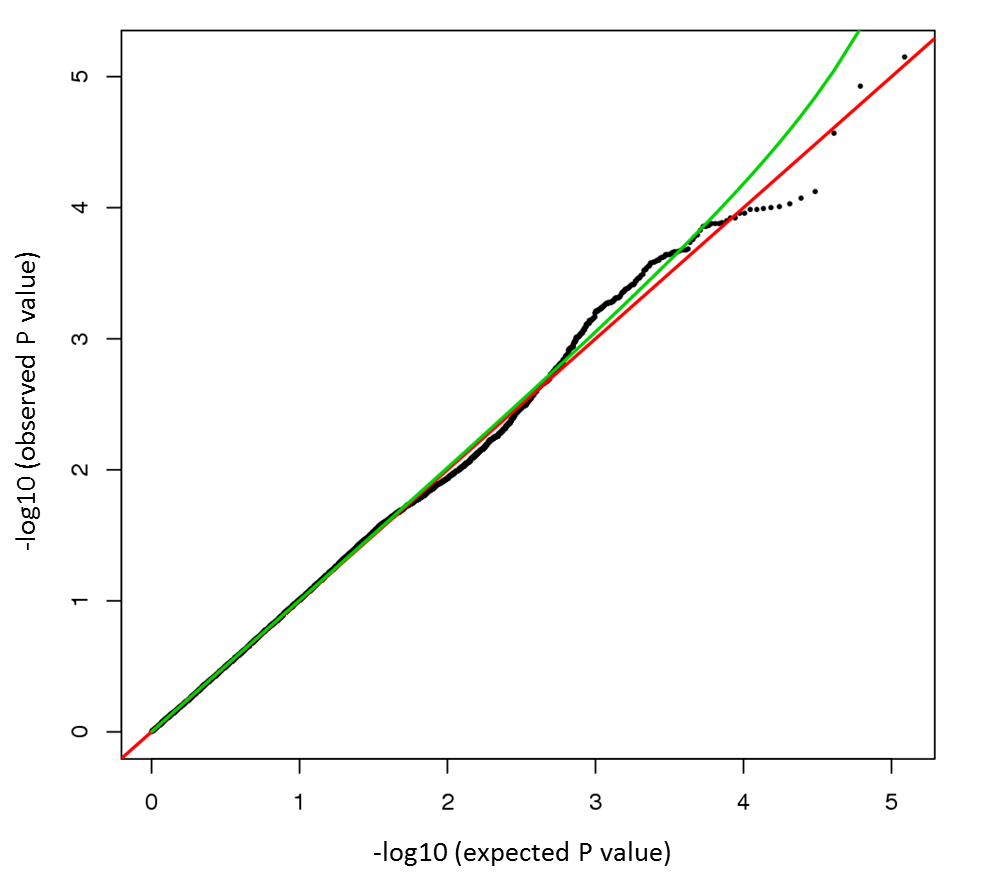

Supplement: S3 Fig — Relationship of observed association statistics with those expected under the null distribution (MAF>2%, Lambda = 1.022). (TIF) [file pone.0155478.s004.tif]

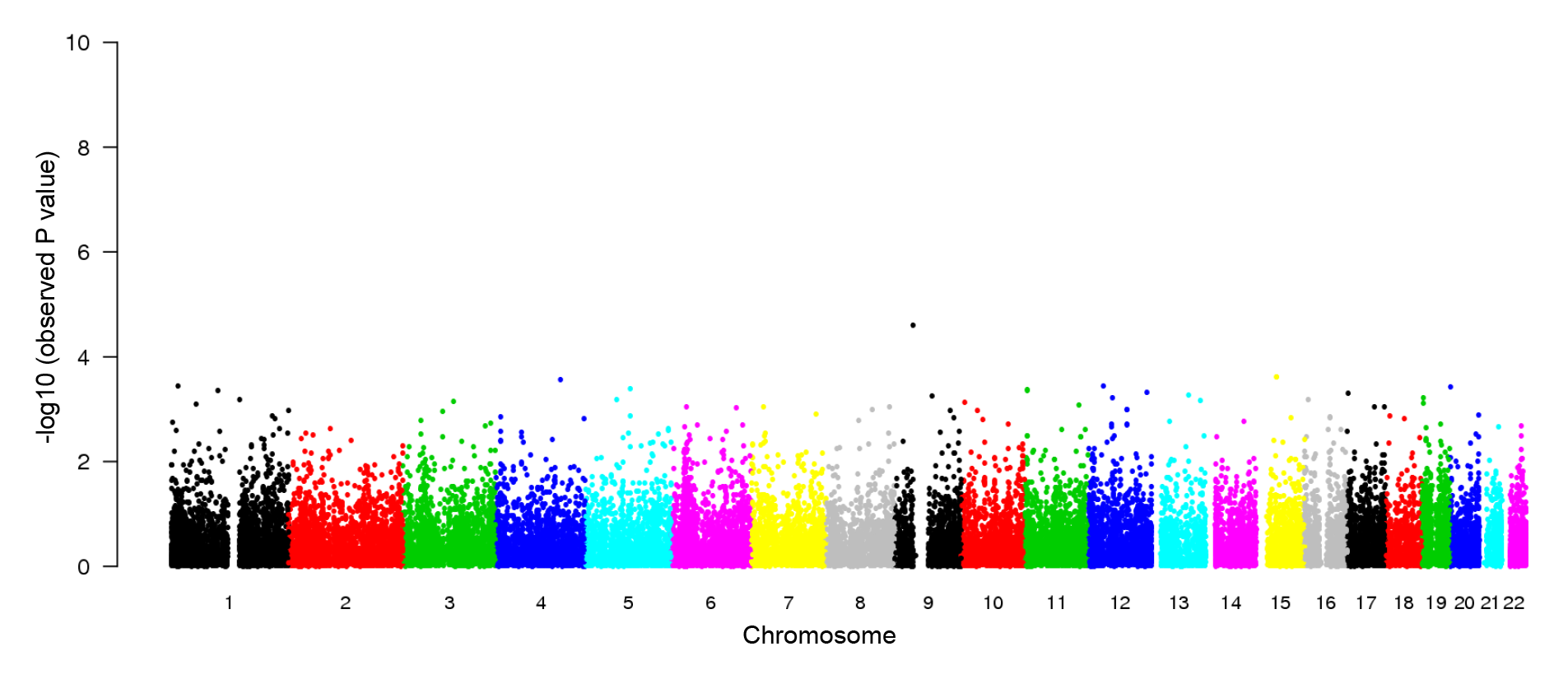

Supplement: S4 Fig — (TIF) [file pone.0155478.s005.tif]

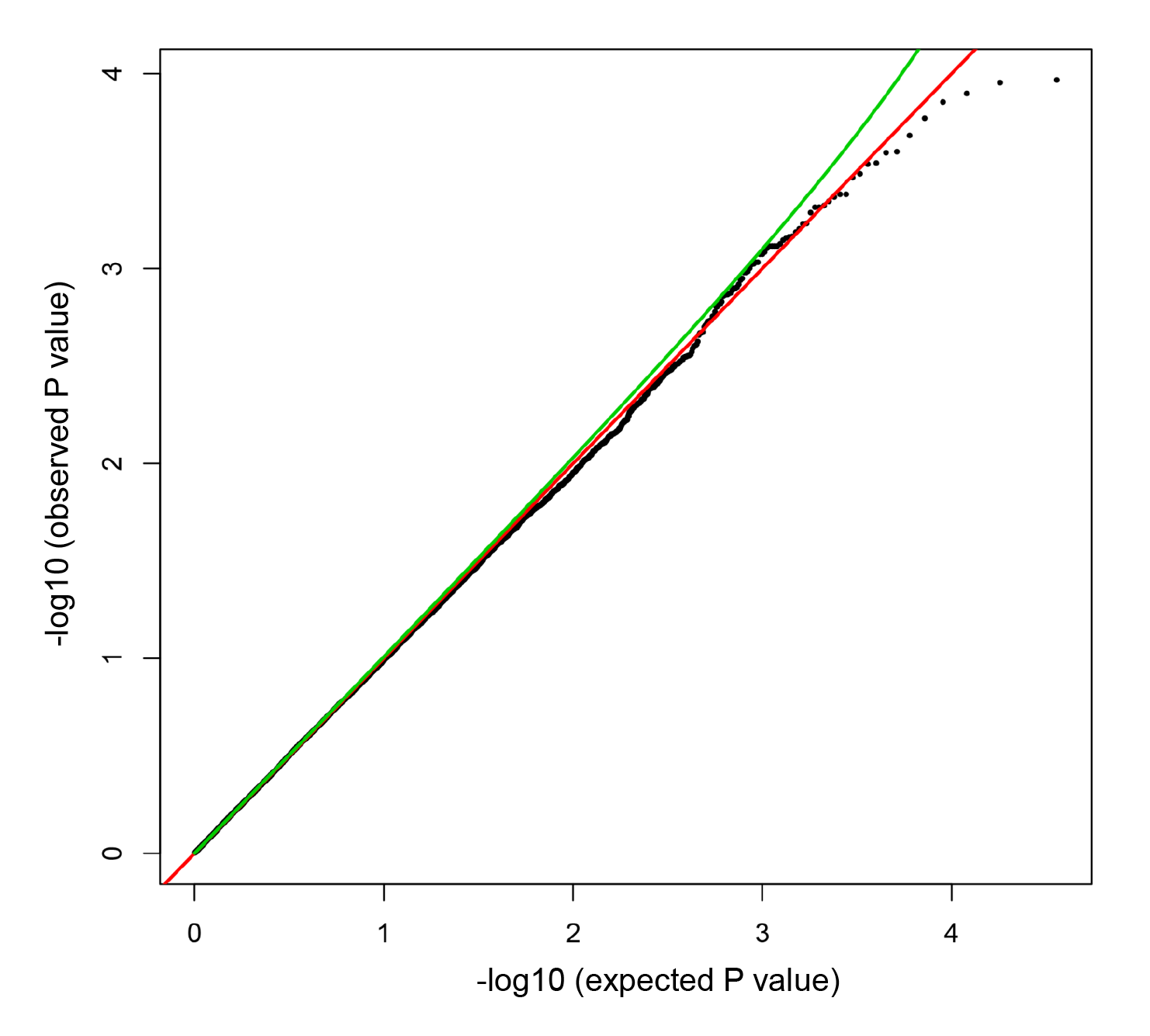

Supplement: S5 Fig — (TIF) [file pone.0155478.s006.tif]

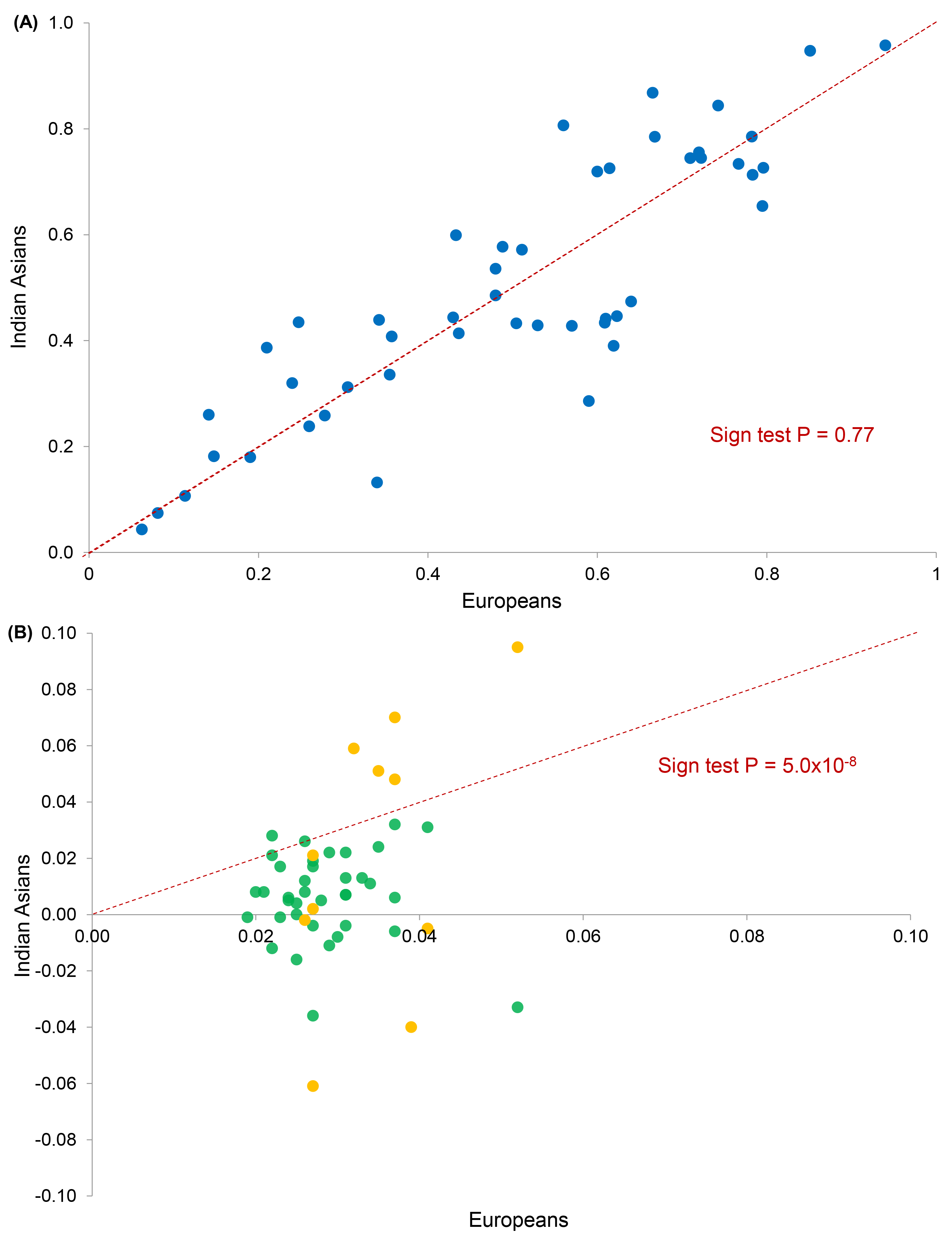

Supplement: S6 Fig — (A) Comparison of risk allele frequencies in South Asians, extended analysis (N = 12,240) and Europeans (reported). (B) Comparison of effect sizes (β WHR) in South Asians and Europeans, extended analysis. Green–men and women combined (37 SNPs in South Asians (N = 12,240) and Europeans (reported)); orange–women alone (11 SNPs in South Asians (N = 2,363) and Europeans (reported)). (TIF) [file pone.0155478.s007.tif]
