# Supplementary material for: Investigation of Genetic Variation Underlying Central Obesity amongst South Asians
Source: PLoS One. 2016 May 19;11(5):e0155478. doi: 10.1371/journal.pone.0155478 (PMC4873263; doi:10.1371/journal.pone.0155478)
Supplement: S1 Table — (DOCX) [file pone.0155478.s008.docx]

**Supplementary Table 1. Characteristics of participants in the genotyping cohorts. Presented as mean (standard deviation), or population %.**

|  | **LOLIPOP Study participants** | | | | | | | | | | | |  | **South Asian replication** | | |
| --- | --- | --- | --- | --- | --- | --- | --- | --- | --- | --- | --- | --- | --- | --- | --- | --- |
|  | **South Asian-GWAS** | | | | |  | **European-GWAS** | | | |  | **South Asian-Exome** |  | **Sikh Diabetes Study** |  | **Mauritius Family Study** |
|  | **South Asian-610** | **South Asian-317** | **South Asian-OmniEE** | **South Asian-P** | **Combined** |  | **European-610** | **European-P** | **European-A** | **Combined** |  |  |  |  |  |  |
|  |  |  |  |  |  |  |  |  |  |  |  |  |  |  |  |  |
| ***n*** | ***6,548*** | ***2,121*** | ***1011*** | ***638*** | ***10,318*** |  | ***927*** | ***644*** | ***582*** | ***2,153*** |  | ***2,637*** |  | ***1,528*** |  | ***394*** |
| Selection criteria | CHD case: control | Population | Population | Population | Mixed |  | Population | CHD case: control | CHD case: control | Mixed |  | Population |  | T2D case: control |  | Families |
|  |  |  |  |  |  |  |  |  |  |  |  |  |  |  |  |  |
| Age (yrs) | 55.4 (10.6) | 48.2 (10.5) | 49.7 (10.0) | 51.1 (8.4) | 53.2 (10.8) |  | 56.0 (9.8) | 55.7 (9.1) | 54.4 (10.4) | 55.5 (9.8) |  | 51.6 (10.1) |  | 53.0 (12.2) |  | 50.3 (13.7) |
| Male (%) | 84.3% | 100.0% | 53.6% | 100.0% | 85.6% |  | 73.1% | 100.0% | 86.9% | 84.9% |  | 68.2% |  | 53.4% |  | 45% |
| CHD (%) | 36.9% | 0.8% | 2.7% | 5.6% | 24.9% |  | 2.4% | 46.6% | 45.5% | 27.3% |  | 6.4% |  | 16.7% |  | - |
| T2D (%) | 22.9% | 17.6% | 22.0% | 18.4% | 21.5% |  | 5.7% | 11.6% | 11.5% | 9.1% |  | 8.7% |  | 52.3% |  | 22.5% |
| Hypertension (%) | 43.4% | 26.1% | 25.7% | 32.1% | 37.7% |  | 21.3% | 37.0% | 36.1% | 30.0% |  | 35.1% |  | 64.2% |  | 24.4% |
|  |  |  |  |  |  |  |  |  |  |  |  |  |  |  |  |  |
| Body mass index (kg/m2) | 27.1 (4.3) | 26.8 (4.3) | 27.7 (4.5) | 27.1 (4.0) | 27.1 (4.3) |  | 27.5 (4.7) | 28.6 (5.3) | 28.4 (4.9) | 28.1 (5.0) |  | 27.6 (4.3) |  | 26.8 (5.0) |  | 25.01 (4.48) |
| **Waist hip ratio** | **0.96 (0.07)** | **0.96 (0.07)** | **0.94 (0.08)** | **0.98 (0.07)** | **0.96 (0.07)** |  | **0.92 (0.08)** | **0.96 (0.07)** | **0.94 (0.08)** | **0.94 (0.08)** |  | **0.95 (0.08)** |  | **0.95 (0.07)** |  | **0.84 (0.07)** |
|  |  |  |  |  |  |  |  |  |  |  |  |  |  |  |  |  |
| Systolic BP (mmHg) | 135.4 (19.5) | 133.5 (20.4) | 128.9 (18.3) | 136.8 (19.4) | 134.5 (19.7) |  | 133.2 (18.9) | 136.7 (19.1) | 135.4 (19.0) | 134.9 (19.1) |  | 132 (19) |  | 138.3 (22,7) |  | 133.9 (24.5) |
| Diastolic BP (mmHg) | 82.0 (10.8) | 82.7 (12.0) | 81.0 (10.7) | 84.9 (11.3) | 82.2 (11.1) |  | 81.1 (10.2) | 81.8 (10.6) | 81.5 (10.6) | 81.4 (10.4) |  | 82 (11) |  | 83.0 (12.9) |  | 78.0 (14.5) |
| Cholesterol (mmol/L) | 5.1 (1.2) | 5.3 (1.1) | 5.3 (1.1) | 5.5 (1.1) | 5.2 (1.1) |  | 5.6 (1.1) | 5.1 (1.1) | 5.2 (1.1) | 5.3 (1.1) |  | 5.3 (1.0) |  | 4.7 (1.3) |  | 5.5 (1.1) |
| LDL cholesterol (mmol/L) | 3.0 (1.0) | 3.3 (0.9) | 3.3 (0.9) | 3.4 (0.9) | 3.1 (1.0) |  | 3.5 (0.9) | 3.1 (0.9) | 3.2 (0.9) | 3.3 (0.9) |  | 3.3 (0.9) |  | 2.8 (1.01) |  | 3.5 (0.9) |
| HDL cholesterol (mmol/L) | 1.2 (0.3) | 1.2 (0.3) | 1.3 (0.3) | 1.2 (0.3) | 1.2 (0.3) |  | 1.4 (0.4) | 1.3 (0.3) | 1.3 (0.3) | 1.3 (0.4) |  | 1.3 (0.3) |  | 1.04 (0.3) |  | 1.2 (0.3) |
| Triglycerides (mmol/L) | 1.8 (1.3) | 1.7 (0.8) | 1.7 (1.2) | 1.9 (1.3) | 1.8 (1.2) |  | 1.5 (1.1) | 1.8 (1.5) | 1.7 (1.2) | 1.7 (1.3) |  | 1.7 (1.1) |  | 2.03 (1.3) |  | 1.7 (1.0) |
| Glucose (mmol/L) | 6.1 (2.3) | 5.9 (2.1) | 5.4 (1.2) | 6.3 (2.5) | 6.0 (2.2) |  | 5.5 (1.6) | 6.0 (2.3) | 5.6 (1.6) | 5.7 (1.8) |  | 5.4 (1.1) |  | 7.5 (3.4) |  | 6.9 (3.4) |
| Insulin (mU/L) | 13.4 (14.4) | 12.6 (9.7) | 13.2 (15.9) | 14.1 (13.5) | 13.3 (13.6) |  | 9.8 (9.0) | 12.5 (14.4) | 11.2 (11.4) | 11.0 (11.5) |  | 12.9 (13.0) |  | 10.9 (13.7) |  | 12.8 (6.8) |
| HbA1c | 6.3 (1.5) | 6.0 (1.4) | 5.8 (0.9) | 5.7 (1.6) | 6.1 (1.4) |  | 5.5 (0.9) | 5.5 (1.3) | 5.3 (1.0) | 5.4 (1.1) |  | 5.7 (0.8) |  | - |  | 5.8 (1.8) |
| HOMA-IR | 3.9 (5.4) | 3.4 (3.5) | 3.2 (5.1) | 4.2 (5.2) | 3.7 (5.0) |  | 2.6 (3.5) | 3.7 (6.3) | 2.9 (3.7) | 3.0 (4.6) |  | 3.2 (4.1) |  | 3.2 (3.5) |  | 3.9 (2.9) |
|  |  |  |  |  |  |  |  |  |  |  |  |  |  |  |  |  |

**Abbreviations: CHD – coronary heart disease; T2D – type-2 diabetes; BP – blood pressure; HOMA-IR – insulin resistance measured by homeostatic model assessment.**

**South Asian- and European-610 – Illumina 610K array; South Asian-317 – Illumina 317K array; South Asian-OmniEE – Illumina OmniExpressExome array; South Asian- and European-P – Perlegen 284K array; European-A – Affymetrix 500 array; South Asian-exome – Illumina HumanExome and Illumina OmniExpressExome arrays; Sikh Diabetes Study – Illumina 660 Quad array; Mauritius Family Study – Illumina HiSeq2000.**
