# Supplementary material for: Investigation of Genetic Variation Underlying Central Obesity amongst South Asians
Source: PLoS One. 2016 May 19;11(5):e0155478. doi: 10.1371/journal.pone.0155478 (PMC4873263; doi:10.1371/journal.pone.0155478)
Supplement: S2 Table — (DOCX) [file pone.0155478.s009.docx]

**Supplementary Table 2. Genotyping cohort platforms and quality metrics.**

| **Cohort** | ***n*** | **GWA platform** | **Genotype calling** | **Sample call rate** | **SNP call rate** | **Lambda §** | **Imputation Software** | **Reference panel*** | **Association software** |
| --- | --- | --- | --- | --- | --- | --- | --- | --- | --- |
|  |  |  |  |  |  |  |  |  |  |
| ***LOLIPOP Study participants*** |  |  |  |  |  |  |  |  |  |
| South Asian-610 | *6,548* | Illumina 610K | BeadStudio | ≥ 0.95 | ≥ 0.98 | 1.032 | Impute 2 | LOLIPOP | SNPTEST |
| South Asian-317 | *2,059* | Illumina 317K | BeadStudio | ≥ 0.95 | ≥ 0.95 | 1.013 | Impute 2 | LOLIPOP | SNPTEST |
| South Asian-OmniEE | *899* | Illumina OmniExpress | Gencall + zCall | ≥ 0.98 | ≥ 0.99 | 0.997 | Impute 2 | LOLIPOP | SNPTEST |
| South Asian-P | *501* | Perlegen 284K | Perlegen | ≥ 0.95 | ≥ 0.95 | 0.987 | Impute 2 | LOLIPOP | SNPTEST |
|  |  |  |  |  |  |  |  |  |  |
| European-610 | *927* | Illumina 610K | BeadStudio | ≥ 0.95 | ≥ 0.95 | 1.001 | Impute 2 | 1000Gv3 | SNPTEST |
| European-P | *644* | Perlegen 284K | Perlegen | ≥ 0.95 | ≥ 0.95 | 0.968 | Impute 2 | 1000Gv3 | SNPTEST |
| European-A | *582* | Affymetrix 500K | BRLMM | ≥ 0.95 | ≥ 0.95 | 0.975 | Impute 2 | 1000Gv3 | SNPTEST |
|  |  |  |  |  |  |  |  |  |  |
| South Asian-exome | *1,664* | Illumina HumanExome | Gencall + zCall | ≥ 0.98 | ≥ 0.99 | 0.975 | n/a | n/a | RAREMETALWORKER |
| South Asian-OmniEE-exome | *977* | Illumina OmniExpressExome | Gencall + zCall | ≥ 0.98 | ≥ 0.99 | 0.967 | n/a | n/a | RAREMETALWORKER |
|  |  |  |  |  |  |  |  |  |  |
| ***South Asian replication*** |  |  |  |  |  |  |  |  |  |
| Sikh Diabetes Study | *1,528* | Illumina 660 Quad | BeadStudio | ≥ 0.95 | ≥ 0.98 | 1.032 | Impute 2 | LOLIPOP | SNPTEST |
| Mauritius Family Study | *394* | Illumina HiSeq2000 | SAMtool | ≥ 0.95 | ≥ 0.98 | 1.032 | ShapeIt2 | 1000Gv3 | SOLAR |
|  |  |  |  |  |  |  |  |  |  |

**Abbreviations : LOLIPOP – whole-genome sequencing data from South Asian participants in the LOLIPOP Study; 1000Gv3 – 1000 genomes phase 1 version 3 (2012).**
