# Supplementary material for: Investigation of Genetic Variation Underlying Central Obesity amongst South Asians
Source: PLoS One. 2016 May 19;11(5):e0155478. doi: 10.1371/journal.pone.0155478 (PMC4873263; doi:10.1371/journal.pone.0155478)
Supplement: S3 Table — (DOCX) [file pone.0155478.s010.docx]

**Supplementary Table 3A: Study power to identify single variants associated with variation in WHR amongst South Asians.**

|  |  |  |  | **Effect size (β WHR)** | | | | | |
| --- | --- | --- | --- | --- | --- | --- | --- | --- | --- |
| **Analysis Strategy** |  | **EAF** |  | **0.075** | **0.10** | **0.15** | **0.25** | **0.5** | **1.0** |
|  |  |  |  |  |  |  |  |  |  |
| **Indian Asian GWAS** |  | **1%** |  | <0.1% | <0.1% | <0.1% | 2.7% | 94.4% | >99.9% |
| ***n*=10,318** |  | **2%** |  | <0.1% | <0.1% | 0.8% | 30.9% | >99.9% | >99.9% |
| **P<5x10-8** |  | **5%** |  | <0.1% | <0.1% | 22.6% | 98.8% | >99.9% | >99.9% |
|  |  | **10%** |  | 1.2% | 11.4% | 84.6% | >99.9% | >99.9% | >99.9% |
|  |  | **25%** |  | 19.6% | 75.1% | >99.9% | >99.9% | >99.9% | >99.9% |
|  |  | **50%** |  | 44.3% | 94.8% | >99.9% | >99.9% | >99.9% | >99.9% |
|  |  |  |  |  |  |  |  |  |  |
| **Indian Asian Exome** |  | **1%** |  | <0.1% | <0.1% | <0.1% | <0.1% | 11.6% | >99.3% |
| ***n*=2,637** |  | **2%** |  | <0.1% | <0.1% | <0.1% | 1.2% | 61.2% | >99.9% |
| **P<1.5x10-6** |  | **5%** |  | <0.1% | <0.1% | 0.1% | 19.8% | >99.9% | >99.9% |
|  |  | **10%** |  | <0.1% | <0.1% | 1.4% | 54.1% | >99.9% | >99.9% |
|  |  | **25%** |  | 0.7% | 4.8% | 23.4% | >99.9% | >99.9% | >99.9% |
|  |  | **50%** |  | 1.9% | 12.0% | 50.4% | >99.9% | >99.9% | >99.9% |
|  |  |  |  |  |  |  |  |  |  |

**Abbreviations: EAF - effect allele frequencies. β WHR per allele copy (adjusted for BMI, inverse normal transformed ranked scale).**

**Supplementary Table 3B: Study power to replicate known WHR SNPs amongst South Asians at P<0.05.**

| **Marker Name** | **Nearest Gene** | **Ref** | **EAF** | **Effect size (β WHR)** | **P value** | **Sample size for 80% power** | **Power in *n*=10,318** | **Power in *n*=1,463 women** |
| --- | --- | --- | --- | --- | --- | --- | --- | --- |
|  |  |  |  |  |  |  |  |  |
| **rs9491696** | ***RSPO3*** | Heid et al. | 0.48 | 0.042 | 1.8E-40 | 8,909 | 84% | NA |
| **rs6905288** | ***VEGFA*** | Heid et al. | 0.56 | 0.036 | 5.9E-25 | 12,286 | 71% | NA |
| **rs984222** | ***TBX15-WARS2*** | Heid et al. | 0.64 | 0.034 | 8.7E-25 | 14,731 | 64% | NA |
| **rs1055144** | ***NFE2L3*** | Heid et al. | 0.21 | 0.040 | 1.0E-24 | 14,781 | 63% | NA |
| **rs10195252** | ***GRB14*** | Heid et al. | 0.60 | 0.033 | 2.1E-24 | 15,012 | 63% | NA |
| **rs4846567** | ***LYPLAL1*** | Heid et al. | 0.72 | 0.034 | 6.9E-21 | 16,836 | 57% | NA |
| **rs1011731** | ***DNM3-PIGC*** | Heid et al. | 0.43 | 0.028 | 9.5E-18 | 20,419 | 50% | NA |
| **rs1294421** | ***LY86*** | Heid et al. | 0.61 | 0.028 | 1.8E-17 | 21,037 | 49% | NA |
| **rs1443512** | ***HOXC13*** | Heid et al. | 0.24 | 0.031 | 6.4E-17 | 22,385 | 47% | NA |
| **rs718314** | ***ITPR2-SSPN*** | Heid et al. | 0.26 | 0.030 | 1.1E-17 | 22,660 | 46% | NA |
| **rs6795735** | ***ADAMTS9*** | Heid et al. | 0.59 | 0.025 | 9.8E-14 | 25,954 | 41% | NA |
| **rs4823006** | ***ZNRF3-KREMEN1*** | Heid et al. | 0.57 | 0.023 | 1.1E-11 | 30,264 | 36% | NA |
| **rs6784615** | ***NISCH-STAB1*** | Heid et al. | 0.94 | 0.043 | 3.8E-10 | 37,628 | 30% | NA |
| **rs6861681** | ***CPEB4*** | Heid et al. | 0.34 | 0.022 | 1.9E-09 | 36,130 | 31% | NA |
| **rs4765219** | ***CCDC92*** | Shungin et al. | 0.67 | 0.028 | 1.6E-15 | 22,636 | 47% | NA |
| **rs979012** | ***BMP2*** | Shungin et al. | 0.34 | 0.027 | 3.3E-14 | 23,986 | 45% | NA |
| **rs17451107** | ***LEKR1*** | Shungin et al. | 0.61 | 0.026 | 1.1E-12 | 24,399 | 45% | NA |
| **rs4081724** | ***CEBPA*** | Shungin et al. | 0.85 | 0.035 | 7.4E-12 | 25,123 | 43% | NA |
| **rs4646404** | ***PEMT*** | Shungin et al. | 0.67 | 0.027 | 1.4E-11 | 24,344 | 45% | NA |
| **rs12679556** | ***MSC*** | Shungin et al. | 0.25 | 0.027 | 2.1E-11 | 28,707 | 39% | NA |
| **rs7759742** | ***BTNL2*** | Shungin et al. | 0.51 | 0.023 | 4.4E-11 | 29,682 | 38% | NA |
| **rs6090583** | ***EYA2*** | Shungin et al. | 0.48 | 0.022 | 6.2E-11 | 32,481 | 35% | NA |
| **rs1440372** | ***SMAD6*** | Shungin et al. | 0.71 | 0.024 | 1.1E-10 | 33,086 | 35% | NA |
| **rs7801581** | ***HOXA11*** | Shungin et al. | 0.24 | 0.027 | 3.7E-10 | 29,510 | 38% | NA |
| **rs1569135** | ***CALCRL*** | Shungin et al. | 0.53 | 0.021 | 5.6E-10 | 35,721 | 32% | NA |
| **rs905938** | ***DCST2*** | Shungin et al. | 0.74 | 0.025 | 7.3E-10 | 32,632 | 35% | NA |
| **rs12608504** | ***JUND*** | Shungin et al. | 0.36 | 0.022 | 8.8E-10 | 35,189 | 33% | NA |
| **rs8042543** | ***KLF13*** | Shungin et al. | 0.78 | 0.026 | 1.2E-09 | 33,827 | 34% | NA |
| **rs1385167** | ***MEIS1*** | Shungin et al. | 0.15 | 0.029 | 1.9E-09 | 36,595 | 31% | NA |
| **rs10919388** | ***GORAB*** | Shungin et al. | 0.72 | 0.024 | 3.2E-09 | 33,792 | 34% | NA |
| **rs10804591** | ***PLXND1*** | Shungin et al. | 0.80 | 0.025 | 6.6E-09 | 39,240 | 30% | NA |
| **rs8030605** | ***RFX7*** | Shungin et al. | 0.14 | 0.030 | 8.8E-09 | 36,213 | 32% | NA |
| **rs10991437** | ***ABCA1*** | Shungin et al. | 0.11 | 0.031 | 1.0E-08 | 41,709 | 28% | NA |
| **rs224333** | ***GDF5*** | Shungin et al. | 0.62 | 0.020 | 2.6E-08 | 41,639 | 29% | NA |
| **rs6556301** | ***FGFR4*** | Shungin et al. | 0.36 | 0.022 | 2.6E-08 | 35,189 | 33% | NA |
| **rs303084** | ***SPATA5-FGF2*** | Shungin et al. | 0.80 | 0.023 | 3.9E-08 | 46,362 | 26% | NA |
| **rs9991328** | ***FAM13A*** | Shungin et al. | 0.49 | 0.019 | 4.5E-08 | 43,498 | 28% | NA |
| **rs11231693** | ***MACROD1-VEGFB*** | Shungin et al. | 0.06 | 0.041 | 4.5E-08 | 41,389 | 29% | NA |
|  |  |  |  |  |  |  |  |  |
|  |  |  |  |  |  |  |  |  |
| **rs4684854** | ***PPARG*** | Randall et al. | 0.43 | 0.037 | 4.2E-14 | 11,692 | NA | 17% |
| **rs10478424** | ***HSD17B4*** | Randall et al. | 0.78 | 0.039 | 3.5E-09 | 15,030 | NA | 14% |
| **rs7830933** | ***NKX2-6*** | Shungin et al. | 0.77 | 0.037 | 1.2E-12 | 16,183 | NA | 13% |
| **rs9687846** | ***MAP3K1*** | Shungin et al. | 0.19 | 0.041 | 3.8E-12 | 15,166 | NA | 14% |
| **rs2925979** | ***CMIP*** | Shungin et al. | 0.31 | 0.032 | 3.4E-11 | 17,913 | NA | 12% |
| **rs12454712** | ***BCL2*** | Shungin et al. | 0.61 | 0.035 | 1.1E-09 | 13,462 | NA | 15% |
| **rs8066985** | ***KCNJ2*** | Shungin et al. | 0.50 | 0.026 | 4.0E-09 | 23,218 | NA | 10% |
| **rs7917772** | ***SFXN2*** | Shungin et al. | 0.62 | 0.027 | 5.5E-09 | 22,845 | NA | 11% |
| **rs1776897** | ***HMGA1*** | Shungin et al. | 0.08 | 0.052 | 6.8E-09 | 19,715 | NA | 12% |
| **rs3805389** | ***NMU*** | Shungin et al. | 0.28 | 0.027 | 4.6E-08 | 26,699 | NA | 10% |
| **rs1534696** | ***SNX10*** | Shungin et al. | 0.44 | 0.027 | 5.4E-08 | 21,844 | NA | 11% |

**Abbreviations: EAF - effect allele frequencies. β WHR per allele copy (adjusted for BMI, inverse normal transformed ranked scale).**
