# Supplementary material for: Investigation of Genetic Variation Underlying Central Obesity amongst South Asians
Source: PLoS One. 2016 May 19;11(5):e0155478. doi: 10.1371/journal.pone.0155478 (PMC4873263; doi:10.1371/journal.pone.0155478)
Supplement: S4 Table — (DOCX) [file pone.0155478.s011.docx]

**Supplementary Table 4A. South Asian GWAS – Top ranking single markers at P<1x10^-5^ under an additive inheritance model.**

|  |  |  |  |  | **SOUTH ASIAN GWAS DISCOVERY** | | | | **SOUTH ASIAN GWAS REPLICATION 1** | | | | **SOUTH ASIAN GWAS REPLICATION 2** | | | | **SOUTH ASIAN GWAS REPLICATION COMBINED** | | | | **SOUTH ASIAN GWAS ALL COMBINED** | | | |
| --- | --- | --- | --- | --- | --- | --- | --- | --- | --- | --- | --- | --- | --- | --- | --- | --- | --- | --- | --- | --- | --- | --- | --- | --- |
| **Marker Name** | **Chr** | **Pos** | **Nearest gene** | **E/A** | **EAF** | **β (SEM)** | ***n*** | **P value** | **EAF** | **β (SEM)** | ***n*** | **P value** | **EAF** | **β (SEM)** | ***n*** | **P value** | **EAF** | **β (SEM)** | ***n*** | **P value** | **EAF** | **β (SEM)** | ***n*** | **P value** |
|  |  |  |  |  |  |  |  |  |  |  |  |  |  |  |  |  |  |  |  |  |  |  |  |  |
| **rs16955700** | 17 | 53242304 | *STXBP4* | C/G | 0.29 | -0.06 (0.01) | 10,004 | 1.8E-06 | 0.26 | 0.04 (0.04) | 1,528 | 3.8E-01 | 0.50 | 0.41 (0.37) | 394 | 2.7E-01 | 0.31 | 0.04 (0.04) | 1,922 | 3.2E-01 | 0.29 | -0.06 (0.01) | 11,926 | 2.3E-05 |
| **rs9600128** | 13 | 74090415 | *KLF12* | A/C | 0.62 | 0.06 (0.01) | 10,005 | 2.0E-06 | 0.35 | -0.03 (0.04) | 1,528 | 4.4E-01 | 0.74 | -0.11 (0.21) | 394 | 6.2E-01 | 0.67 | 0.03 (0.04) | 1,922 | 5.1E-01 | 0.63 | 0.06 (0.01) | 11,927 | 2.5E-06 |
| **rs61819000** | 1 | 191852635 | *RGS18* | A/G | 0.06 | -0.14 (0.03) | 9,505 | 2.3E-06 | 0.07 | 0.05 (0.09) | 1,528 | 5.9E-01 | 0.50 | -0.03 (0.53) | 394 | 9.5E-01 | 0.16 | 0.05 (0.09) | 1,922 | 6.1E-01 | 0.08 | -0.12 (0.03) | 11,427 | 1.5E-05 |
| **rs6841445** | 4 | 13127303 | *RAB28* | T/C | 0.54 | -0.07 (0.02) | 9,505 | 2.6E-06 | 0.45 | -0.04 (0.05) | 1,528 | 4.3E-01 | 0.67 | -0.01 (0.19) | 394 | 9.6E-01 | 0.58 | 0.04 (0.05) | 1,922 | 4.7E-01 | 0.55 | -0.06 (0.02) | 11,427 | 2.3E-05 |
| **rs76871033** | 9 | 113104907 | *TXNDC8* | T/C | 0.97 | -0.25 (0.05) | 9,505 | 3.3E-06 | 0.02 | -0.21 (0.18) | 1,528 | 2.7E-01 | NA | NA | NA | NA | 0.98 | 0.20 (0.18) | 1,528 | 2.7E-01 | 0.97 | -0.21 (0.05) | 11,033 | 3.0E-05 |
| **rs1215050** | 4 | 98849826 | *STPG2* | A/G | 0.60 | -0.06 (0.01) | 10,005 | 4.2E-06 | 0.38 | 0.01 (0.04) | 1,528 | 8.7E-01 | 0.62 | -0.20 (0.23) | 394 | 3.8E-01 | 0.62 | -0.01 (0.04) | 1,922 | 7.7E-01 | 0.60 | -0.05 (0.01) | 11,927 | 8.2E-06 |
| **rs34182841** | 3 | 99091120 | *COL8A1* | T/C | 0.07 | 0.12 (0.03) | 10,005 | 4.8E-06 | 0.07 | -0.09 (0.08) | 1,528 | 2.6E-01 | 0.25 | -1.08 (0.92) | 394 | 2.4E-01 | 0.11 | -0.10 (0.08) | 1,922 | 2.2E-01 | 0.07 | 0.10 (0.03) | 11,927 | 7.4E-05 |
| **rs11625034** | 14 | 54349976 | *BMP4* | A/G | 0.33 | 0.08 (0.02) | 9,505 | 5.0E-06 | 0.27 | 0.05 (0.06) | 1,528 | 3.5E-01 | 0.35 | -0.45 (0.27) | 394 | 9.7E-02 | 0.29 | 0.03 (0.06) | 1,922 | 5.7E-01 | 0.32 | 0.07 (0.02) | 11,427 | 5.9E-06 |
| **rs57561811** | 14 | 61572178 | *SLC38A6* | T/C | 0.75 | -0.07 (0.02) | 10,004 | 5.0E-06 | 0.23 | 0.05 (0.05) | 1,528 | 3.1E-01 | 0.53 | -0.27 (0.22) | 394 | 2.1E-01 | 0.72 | -0.06 (0.05) | 1,922 | 2.1E-01 | 0.74 | -0.07 (0.01) | 11,926 | 2.3E-06 |
| **rs17062295** | 18 | 72065084 | *FAM69C* | A/G | 0.80 | 0.07 (0.02) | 10,004 | 6.1E-06 | 0.19 | 0.04 (0.05) | 1,528 | 4.3E-01 | 0.80 | -0.68 (0.65) | 394 | 3.0E-01 | 0.81 | -0.04 (0.05) | 1,922 | 3.9E-01 | 0.80 | 0.06 (0.02) | 11,926 | 5.3E-05 |
| **rs139256956** | 19 | 31097322 | *ZNF536* | A/C | 0.98 | -0.24 (0.05) | 9,505 | 6.2E-06 | 0.02 | 0.32 (0.17) | 1,528 | 6.4E-02 | 0.63 | -0.28 (0.53) | 394 | 5.9E-01 | 0.91 | -0.32 (0.17) | 1,922 | 6.7E-02 | 0.97 | -0.25 (0.05) | 11,427 | 1.1E-06 |
| **rs11160136** | 14 | 94196116 | *PRIMA1* | T/G | 0.24 | 0.08 (0.02) | 9,504 | 6.5E-06 | 0.23 | -0.01 (0.06) | 1,528 | 8.7E-01 | 0.19 | -0.30 (0.16) | 394 | 6.1E-02 | 0.22 | -0.04 (0.05) | 1,922 | 4.5E-01 | 0.24 | 0.07 (0.02) | 10,627 | 6.8E-05 |
| **rs60338893** | 6 | 124983468 | *NKAIN2* | T/C | 0.76 | 0.06 (0.01) | 10,005 | 7.6E-06 | 0.24 | 0.04 (0.04) | 1,528 | 3.6E-01 | 0.58 | 0.36 (0.41) | 394 | 3.8E-01 | 0.72 | -0.03 (0.04) | 1,922 | 4.1E-01 | 0.75 | 0.05 (0.01) | 11,927 | 7.4E-05 |
| **rs117898805** | 7 | 54516128 | *VSTM2A* | T/G | 0.92 | -0.11 (0.02) | 10,005 | 7.6E-06 | 0.08 | 0.07 (0.08) | 1,528 | 3.6E-01 | 0.50 | 0.78 (0.92) | 394 | 3.9E-01 | 0.84 | -0.07 (0.08) | 1,922 | 4.0E-01 | 0.91 | -0.11 (0.02) | 11,927 | 6.4E-06 |
| **rs78656316** | 15 | 38471754 | *SPRED1* | T/G | 0.97 | 0.24 (0.05) | 9,505 | 8.9E-06 | 0.02 | 0.39 (0.20) | 1,528 | 5.0E-02 | NA | NA | NA | NA | 0.98 | -0.39 (0.20) | 1,528 | 5.0E-02 | 0.97 | 0.19 (0.05) | 11,033 | 1.6E-04 |
| **rs4725192** | 7 | 9426583 | *NXPH1* | T/G | 0.82 | 0.07 (0.02) | 9,504 | 9.7E-06 | 0.20 | -0.05 (0.05) | 1,528 | 2.9E-01 | 0.75 | -0.01 (0.19) | 394 | 9.6E-01 | 0.79 | 0.05 (0.05) | 1,922 | 3.3E-01 | 0.81 | 0.07 (0.02) | 11,426 | 6.8E-06 |

**Discovery – LOLIPOP Study; Replication 1 – Sikh Diabetes Study (25); Replication 2 – Mauritius Study (28).**

**Abbreviations: Chr - chromosome; Pos - position; E/A – effect and alternative alleles; EAF – effect allele frequencies; β (SEM) – β coefficients (standard error of mean) per change of WHR-increasing allele on WHR (adjusted for BMI, inverse normal transformed ranked scale); *n* – number of participants; P value – for association with WHR.**

**Supplementary Table 4B. South Asian GWAS – Replication in Europeans of top ranking single markers at P<1x10^-5^ under an additive inheritance model.**

|  |  |  |  |  | **SOUTH ASIAN GWAS (DISCOVERY)** | | | | | | | | **EUROPEAN REPORTED (REPLICATION)** | | | | | | | | | | | | | |  |  |  |
| --- | --- | --- | --- | --- | --- | --- | --- | --- | --- | --- | --- | --- | --- | --- | --- | --- | --- | --- | --- | --- | --- | --- | --- | --- | --- | --- | --- | --- | --- |
| **MarkerName** | **Chr** | **Pos** | **Nearest gene** | **E/A** | **EAF** | | **β (SEM) WHR** | | ***n*** | | **P value** | | **Lead SNP** | | **R^2^** | | **E/A** | | **EAF** | **β (SEM) WHR** | | | | ***n*** | | **P value** |  |  |  |
|  |  |  |  |  |  | |  | |  | |  | |  | |  | |  | |  |  | | | |  | |  |  |  |  |
| **rs16955700** | 17 | 53242304 | *STXBP4* | C/G | 0.29 | | -0.06 (0.01) | | 10,004 | | 1.8E-06 | | rs16955700 | | 1.0 | | C/G | | . | 0.01 (0.02) | | | | 4,091 | | 4.6E-01 |  |  |  |
| **rs9600128** | 13 | 74090415 | *KLF12* | A/C | 0.62 | | 0.06 (0.01) | | 10,005 | | 2.0E-06 | | rs9600128 | | 1.0 | | A/C | | 0.81 | 0.01 (0.01) | | | | 77,336 | | 3.1E-01 |  |  |  |
| **rs61819000** | 1 | 191852635 | *RGS18* | A/G | 0.06 | | -0.14 (0.03) | | 9,505 | | 2.3E-06 | | NA | | NA | | NA | | NA | NA | | | | NA | | NA |  |  |  |
| **rs6841445** | 4 | 13127303 | *RAB28* | T/C | 0.54 | | -0.07 (0.02) | | 9,505 | | 2.6E-06 | | rs6841445 | | 1.0 | | T/C | | 0.65 | -0.01 (0.01) | | | | 73,019 | | 1.5E-01 |  |  |  |
| **rs76871033** | 9 | 113104907 | *TXNDC8* | T/C | 0.97 | | -0.25 (0.05) | | 9,505 | | 3.3E-06 | | NA | | NA | | NA | | NA | NA | | | | NA | | NA |  |  |  |
| **rs1215050** | 4 | 98849826 | *STPG2* | A/G | 0.6 | | -0.06 (0.01) | | 10,005 | | 4.2E-06 | | rs1215050 | | 1.0 | | A/G | | 0.55 | 0.00 (0.00) | | | | 77,335 | | 5.2E-01 |  |  |  |
| **rs34182841** | 3 | 99091120 | *COL8A1* | T/C | 0.07 | | 0.12 (0.03) | | 10,005 | | 4.8E-06 | | rs10935768 | | 1.0 | | A/C | | 0.09 | 0.01 (0.01) | | | | 73,037 | | 3.7E-01 |  |  |  |
| **rs11625034** | 14 | 54349976 | *BMP4* | A/G | 0.33 | | 0.08 (0.02) | | 9,505 | | 5.0E-06 | | rs11625034 | | 1.0 | | A/G | | 0.43 | 0.01 (0.01) | | | | 77,306 | | 7.6E-02 |  |  |  |
| **rs57561811** | 14 | 61572178 | *SLC38A6* | T/C | 0.75 | | -0.07 (0.02) | | 10,004 | | 5.0E-06 | | NA | | NA | | NA | | NA | NA | | | | NA | | NA |  |  |  |
| **rs17062295** | 18 | 72065084 | *FAM69C* | A/G | 0.8 | | 0.07 (0.02) | | 10,004 | | 6.1E-06 | | rs17062295 | | 1.0 | | A/G | | . | 0.01 (0.02) | | | | 4,022 | | 7.2E-01 |  |  |  |
| **rs139256956** | 19 | 31097322 | *ZNF536* | A/C | 0.98 | | -0.24 (0.05) | | 9,505 | | 6.2E-06 | | NA | | NA | | NA | | NA | NA | | | | NA | | NA |  |  |  |
| **rs11160136** | 14 | 94196116 | *PRIMA1* | T/G | 0.24 | | 0.08 (0.02) | | 9,504 | | 6.5E-06 | | rs8015340 | | 0.6 | | A/G | | 0.48 | 0.00 (0.01) | | | | 73,019 | | 7.0E-01 |  |  |  |
| **rs60338893** | 6 | 124983468 | *NKAIN2* | T/C | 0.76 | | 0.06 (0.01) | | 10,005 | | 7.6E-06 | | NA | | NA | | NA | | NA | NA | | | | NA | | NA |  |  |  |
| **rs117898805** | 7 | 54516128 | *VSTM2A* | T/G | 0.92 | | -0.11 (0.02) | | 10,005 | | 7.6E-06 | | rs17172341 | | 1.0 | | T/C | | 0.02 | 0.00 (0.01) | | | | 74,115 | | 9.1E-01 |  |  |  |
| **rs78656316** | 15 | 38471754 | *SPRED1* | T/G | 0.97 | | 0.24 (0.05) | | 9,505 | | 8.9E-06 | | NA | | NA | | NA | | NA | NA | | | | NA | | NA |  |  |  |
| **rs4725192** | 7 | 9426583 | *NXPH1* | T/G | 0.82 | | 0.07 (0.02) | | 9,504 | | 9.7E-06 | | rs7786511 | | 1.0 | | T/G | | 0.83 | 0.00 (0.01) | | | | 77,336 | | 6.3E-01 |  |  |  |
|  |  |  |  |  |  |  | |  | |  | |  | |  | |  | |  | | |  |  |  | |  | | |  |  |

**Discovery – LOLIPOP Study; Replication – Europeans, reported GIANT consortium meta-analysis data (20).**

**Abbreviations: Chr - chromosome; Pos - position; E/A – effect and alternative alleles; EAF – effect allele frequencies; β (SEM) – β coefficients (standard error of mean) per change of WHR-increasing allele on WHR (adjusted for BMI, inverse normal transformed ranked scale); Het P value – for heterogeneity in the meta-analysis; *n* – number of participants; P value – for association with WHR; Lead SNP – representative SNP at the discovery locus used in the replication analysis; R^2^ – linkage disequilibrium between lead and discovery SNP.**

**Supplementary Table 4C. South Asian GWAS – Top ranking single markers at P<1x10^-5^ under dominant and recessive inheritance models.**

|  |  |  |  |  |  |  | **SOUTH ASIAN GWAS DISCOVERY** | | | | |
| --- | --- | --- | --- | --- | --- | --- | --- | --- | --- | --- | --- |
| **Model** | **MarkerName** | **Chr** | **Pos** | **Nearest gene** | **E/A** |  | **EAF** | **β (SEM) WHR** | **Het P val** | ***n*** | **P value** |
|  |  |  |  |  |  |  |  |  |  |  |  |
| **Dominant** | **rs197160** | 19 | 4981325 | *KDM4B* | T/C |  | 0.77 | -0.22 (0.04) | 2.5E-02 | 10,004 | 1.2E-07 |
|  | **rs7131566** | 11 | 86604996 | *PRSS23* | A/T |  | 0.19 | -0.22 (0.05) | 1.1E-01 | 10,005 | 8.3E-07 |
|  | **rs9600128** | 13 | 74090415 | *KLF12* | A/C |  | 0.62 | 0.09 (0.02) | 5.1E-01 | 10,005 | 1.4E-06 |
|  | **rs1304941** | 6 | 124996466 | *NKAIN2* | T/C |  | 0.24 | -0.08 (0.02) | 1.5E-01 | 10,005 | 2.1E-06 |
|  | **rs1215050** | 4 | 98849826 | *STPG2* | A/G |  | 0.60 | -0.08 (0.02) | 7.3E-01 | 10,005 | 2.1E-06 |
|  | **rs4766971** | 12 | 113018479 | *PTPN11* | A/G |  | 0.14 | 0.32 (0.07) | 4.3E-01 | 10,005 | 2.7E-06 |
|  | **rs6658680** | 1 | 4915470 | *AJAP1* | A/G |  | 0.36 | 0.13 (0.03) | 6.2E-01 | 10,004 | 3.2E-06 |
|  | **rs3745080** | 18 | 73143207 | *SMIM21* | T/C |  | 0.89 | -0.34 (0.07) | 3.4E-01 | 10,005 | 3.6E-06 |
|  | **rs76213488** | 4 | 110232411 | *COL25A1* | A/G |  | 0.09 | 0.46 (0.1) | 5.7E-03 | 10,004 | 3.6E-06 |
|  | **rs12505856** | 4 | 174989417 | *FBXO8* | T/C |  | 0.36 | -0.08 (0.02) | 6.6E-01 | 10,004 | 4.5E-06 |
|  | **rs11868244** | 17 | 34243343 | *RDM1* | T/C |  | 0.88 | -0.32 (0.07) | 8.4E-01 | 10,004 | 4.6E-06 |
|  | **rs34182841** | 3 | 99091120 | *COL8A1* | T/C |  | 0.07 | 0.13 (0.03) | 9.0E-01 | 10,005 | 4.6E-06 |
|  | **rs17171803** | 7 | 41054134 | *SUGCT* | C/G |  | 0.17 | -0.24 (0.05) | 1.5E-01 | 10,005 | 4.7E-06 |
|  | **rs4099289** | 16 | 74468856 | *GLG1* | A/G |  | 0.28 | 0.14 (0.03) | 2.5E-01 | 10,004 | 6.8E-06 |
|  | **rs866510** | 12 | 25240964 | *LRMP* | C/G |  | 0.23 | -0.17 (0.04) | 8.0E-01 | 10,004 | 9.2E-06 |
|  | **rs13254852** | 8 | 93436894 | *RUNX1T1* | T/C |  | 0.20 | 0.08 (0.02) | 1.7E-01 | 10,004 | 9.7E-06 |
|  |  |  |  |  |  |  |  |  |  |  |  |
| **Recessive** | **rs17149491** | 11 | 86606059 | *PRSS23* | A/G |  | 0.81 | 0.22 (0.05) | 1.1E-01 | 10,005 | 7.9E-07 |
|  | **rs60338893** | 6 | 124983468 | *NKAIN2* | T/C |  | 0.76 | 0.08 (0.02) | 1.7E-01 | 10,005 | 1.2E-06 |
|  | **rs504071** | 4 | 13127578 | *RAB28* | A/G |  | 0.48 | 0.09 (0.02) | 3.0E-01 | 10,005 | 3.0E-06 |
|  | **rs11653282** | 17 | 34236746 | *RDM1* | T/C |  | 0.12 | 0.32 (0.07) | 8.5E-01 | 10,005 | 3.6E-06 |
|  | **rs1839903** | 4 | 98791414 | *STPG2* | T/C |  | 0.60 | -0.08 (0.02) | 8.5E-01 | 10,005 | 4.1E-06 |
|  | **rs10085962** | 8 | 93431643 | *RUNX1T1* | A/G |  | 0.18 | 0.09 (0.02) | 1.5E-01 | 10,005 | 4.3E-06 |
|  | **rs4559467** | 1 | 4925492 | *AJAP1* | A/G |  | 0.63 | -0.12 (0.03) | 5.4E-01 | 10,005 | 4.3E-06 |
|  | **rs60369504** | 4 | 110234525 | *COL25A1* | T/C |  | 0.09 | 0.45 (0.1) | 9.1E-03 | 10,005 | 4.8E-06 |
|  | **rs10486701** | 7 | 41051036 | *SUGCT* | A/G |  | 0.83 | 0.23 (0.05) | 1.5E-01 | 10,005 | 4.8E-06 |
|  | **rs10012956** | 4 | 174989068 | *FBXO8* | C/G |  | 0.36 | -0.08 (0.02) | 6.6E-01 | 10,004 | 5.1E-06 |
|  | **rs13071410** | 3 | 99093002 | *COL8A1* | T/C |  | 0.95 | -0.13 (0.03) | 8.8E-01 | 10,005 | 6.4E-06 |
|  | **rs2072366** | 17 | 48752890 | *ABCC3* | A/G |  | 0.76 | -0.19 (0.04) | 6.9E-02 | 10,004 | 7.3E-06 |
|  | **rs7972080** | 12 | 25239970 | *LRMP* | T/C |  | 0.22 | -0.17 (0.04) | 7.9E-01 | 10,005 | 7.8E-06 |
|  | **rs2247784** | 18 | 44746997 | *SKOR2* | A/G |  | 0.42 | 0.08 (0.02) | 3.0E-01 | 10,005 | 8.3E-06 |
|  | **rs2413250** | 22 | 35044299 | *ISX* | A/T |  | 0.93 | -0.57 (0.13) | 7.3E-04 | 10,004 | 8.3E-06 |
|  | **rs58998439** | 3 | 144600653 | *SLC9A9* | A/G |  | 0.80 | -0.19 (0.04) | 2.1E-01 | 10,005 | 8.9E-06 |
|  | **rs4586589** | 2 | 192417747 | *NABP1* | A/G |  | 0.46 | -0.08 (0.02) | 4.2E-01 | 10,005 | 1.0E-05 |
|  |  |  |  |  |  |  |  |  |  |  |  |

**Discovery – LOLIPOP Study.**

**Abbreviations: Chr - chromosome; Pos – position; E/A – effect and alternative alleles; EAF – effect allele frequencies; β (SEM) – β coefficients (standard error of mean) per change of WHR-increasing allele on WHR (adjusted for BMI, inverse normal transformed ranked scale); Het P value – for heterogeneity in the meta-analysis; *n* – number of participants; P value – for association with WHR.**
