# Supplementary material for: Investigation of Genetic Variation Underlying Central Obesity amongst South Asians
Source: PLoS One. 2016 May 19;11(5):e0155478. doi: 10.1371/journal.pone.0155478 (PMC4873263; doi:10.1371/journal.pone.0155478)
Supplement: S5 Table — (DOCX) [file pone.0155478.s012.docx]

**Supplementary Table 5. South Asian GWAS targeted analysis – Top ranking population-specific markers under an additive inheritance model.**

|  |  |  |  |  |  | **SOUTH ASIAN GWAS DISCOVERY** | | | |
| --- | --- | --- | --- | --- | --- | --- | --- | --- | --- |
| **SNP selection / threshold P value** | **Marker Name** | **Chr** | **Pos** | **E/A** |  | **EAF** | **β (SEM)** | ***n*** | **P value** |
|  |  |  |  |  |  |  |  |  |  |
| **EAF >2%** | rs78930663 | 15 | 53746073 | C/G |  | 0.03 | 0.368 (0.082) | 7047 | 1.1E-05 |
| **N=122,391** | rs72938326 | 3 | 99067392 | C/T |  | 0.03 | 0.208 (0.047) | 9505 | 1.2E-05 |
| **P<4.0x10^-7^** | 3:189943605 | 3 | 189943605 | A/T |  | 0.06 | -0.161 (0.038) | 9505 | 2.7E-05 |
|  | rs116833927 | 6 | 150943097 | C/T |  | 0.03 | 0.198 (0.019) | 10005 | 7.5E-05 |
|  | rs150518978 | 17 | 35658372 | A/G |  | 0.03 | 0.141 (0.036) | 10005 | 8.5E-05 |
|  |  |  |  |  |  |  |  |  |  |
| **EAF >5%** | 3:189943605 | 3 | 189943605 | A/T |  | 0.06 | -0.161 (0.038) | 9505 | 2.7E-05 |
| **N=38,639** | 3:38306865 | 3 | 38306865 | A/G |  | 0.05 | 0.155 (0.040) | 10005 | 1.0E-04 |
| **P<1.3x10^-6^** | rs142111863 | 3 | 138415926 | G/A |  | 0.05 | 0.173 (0.046) | 9106 | 1.5E-04 |
|  | rs11020147 | 11 | 92750693 | C/T |  | 0.11 | -0.120 (0.032) | 9505 | 1.8E-04 |
|  | rs74006515 | 16 | 5982389 | A/G |  | 0.11 | 0.092 (0.025) | 9505 | 2.1E-04 |
|  |  |  |  |  |  |  |  |  |  |
| **EAF >10%** | rs11020147 | 11 | 92750693 | C/T |  | 0.11 | -0.120 (0.032) | 9505 | 1.8E-04 |
| **N=7,349** | rs74006515 | 16 | 5982389 | A/G |  | 0.11 | 0.092 (0.025) | 9505 | 2.1E-04 |
| **P<6.8x10^-6^** | rs76170449 | 4 | 110289592 | A/T |  | 0.10 | 0.092 (0.025) | 10005 | 2.2E-04 |
|  | rs187727569 | 9 | 112110215 | C/T |  | 0.15 | -0.103 (0.030) | 9505 | 6.9E-04 |
|  | rs6978502 | 7 | 50331632 | A/G |  | 0.15 | -0.080 (0.025) | 9504 | 1.3E-03 |
|  |  |  |  |  |  |  |  |  |  |
| **EAF >20%** | rs141848552 | 2 | 69802633 | T/C |  | 0.22 | 0.042 (0.018) | 10005 | 1.8E-02 |
| **N=596** | rs76205452 | 9 | 119247409 | G/A |  | 0.31 | -0.030 (0.015) | 10005 | 4.6E-02 |
| **P<8.4x10^-5^** | 3:44250057 | 3 | 44250057 | G/T |  | 0.24 | -0.060 (0.030) | 6546 | 4.7E-02 |
|  | rs75091040 | 20 | 17799536 | A/C |  | 0.23 | 0.034 (0.017) | 10004 | 4.7E-02 |
|  | rs117125185 | 11 | 88276962 | G/T |  | 0.20 | -0.041 (0.023) | 9504 | 7.8E-02 |

**Abbreviations: Chr – chromosome; Pos – position; E/A – effect and alternative alleles; EAF – effect allele frequencies; β WHR – β coefficients per change of WHR-increasing allele on WHR (adjusted for BMI, inverse normal transformed ranked scale); Het P value – for heterogeneity in the meta-analysis; *n* – number of participants; P value – for association with WHR.**
