# Supplementary material for: Investigation of Genetic Variation Underlying Central Obesity amongst South Asians
Source: PLoS One. 2016 May 19;11(5):e0155478. doi: 10.1371/journal.pone.0155478 (PMC4873263; doi:10.1371/journal.pone.0155478)
Supplement: S6 Table — (DOCX) [file pone.0155478.s013.docx]

**Supplementary Table 6A. South Asian exome-array analysis –** **Top ranking single markers at P<1x10^-3^.**

|  |  |  |  |  |  | **SOUTH ASIAN EXOME (DISCOVERY)** | | | | |
| --- | --- | --- | --- | --- | --- | --- | --- | --- | --- | --- |
| **MarkerName** | **Chr** | **Pos** | **Nearest Gene** | **E/A** |  | **EAF** | **β WHR** | **Het P Val** | ***n*** | **P value** |
|  |  |  |  |  |  |  |  |  |  |  |
| rs34996750 | 18 | 9256258 | *ANKRD12* | T/C |  | 0.98 | 0.406 | 7.9E-01 | 2637 | 1.1E-04 |
| rs59550885 | 15 | 43571958 | *TGM7* | C/A |  | 0.02 | -0.431 | 5.6E-02 | 2637 | 1.1E-04 |
| rs2018601 | 4 | 136694463 | *PABPC4L* | T/C |  | 0.53 | 0.105 | 6.3E-01 | 2637 | 1.3E-04 |
| rs2149547 | 9 | 37990801 | *SHB* | G/A |  | 0.40 | 0.107 | 7.9E-01 | 2637 | 1.4E-04 |
| rs1236913 | 9 | 125133479 | *PTGS1* | T/C |  | 0.19 | -0.128 | 5.5E-01 | 2637 | 2.1E-04 |
| rs142639450 | 20 | 61451333 | *COL9A3* | G/A |  | 0.04 | 0.254 | 6.2E-01 | 2637 | 2.5E-04 |
| rs8147 | 3 | 186301703 | *DNAJB11* | A/G |  | 0.75 | -0.116 | 8.8E-01 | 2637 | 2.9E-04 |
| rs10511089 | 3 | 84481068 | *CADM2* | G/T |  | 0.10 | 0.168 | 4.9E-01 | 2637 | 3.3E-04 |
| rs12638212 | 3 | 108159977 | *MYH15* | G/A |  | 0.16 | 0.134 | 8.8E-01 | 2637 | 3.4E-04 |
| rs34123504 | 11 | 118771747 | *BCL9L* | A/G |  | 0.98 | -0.351 | 3.8E-01 | 2637 | 4.2E-04 |
| rs3102817 | 7 | 68544052 | *AUTS2* | C/A |  | 0.39 | -0.099 | 3.7E-01 | 2637 | 4.3E-04 |
| rs498573 | 11 | 128054642 | *ETS1* | C/T |  | 0.74 | 0.112 | 2.6E-01 | 2637 | 4.5E-04 |
| rs34458979 | 18 | 41148471 | *SETBP1* | T/C |  | 0.71 | -0.106 | 8.2E-02 | 2637 | 4.8E-04 |
| rs12712508 | 2 | 36771309 | *CRIM1* | A/G |  | 0.70 | 0.103 | 1.2E-01 | 2637 | 4.9E-04 |
| rs2228305 | 17 | 80042792 | *FASN* | C/T |  | 0.08 | -0.172 | 1.7E-01 | 2637 | 4.9E-04 |
| rs890947 | 5 | 66538400 | *CD180* | G/A |  | 0.58 | 0.095 | 1.1E-01 | 2637 | 5.2E-04 |
| rs200294583 | 12 | 50100925 | *FMNL3* | C/A |  | 0.01 | 0.709 | NA | 977 | 5.9E-04 |
| rs73038948 | 19 | 39228244 | *CAPN12* | T/C |  | 0.84 | -0.131 | 4.5E-01 | 2637 | 5.9E-04 |
| rs139962539 | 1 | 11102940 | *MASP2* | G/A |  | 0.02 | 0.322 | 5.4E-01 | 2637 | 6.3E-04 |
| rs17778003 | 8 | 135669810 | *ZFAT* | C/T |  | 0.06 | 0.194 | 1.2E-01 | 2637 | 6.5E-04 |
| rs2273510 | 6 | 139097232 | *CCDC28A* | C/T |  | 0.03 | -0.294 | 9.8E-01 | 2637 | 6.9E-04 |
| rs3085 | 1 | 207857254 | *CR1L* | A/G |  | 0.77 | -0.110 | 2.0E-01 | 2637 | 6.9E-04 |
| rs6794 | 3 | 45052775 | *EXOSC7* | G/C |  | 0.72 | -0.101 | 9.8E-01 | 2637 | 7.2E-04 |
| rs138658798 | 10 | 7605243 | *ITIH5* | C/T |  | 0.02 | -0.356 | 7.5E-01 | 2637 | 7.7E-04 |
| rs57971665 | 12 | 80878317 | *PTPRQ* | G/A |  | 0.02 | 0.346 | 3.2E-01 | 2637 | 7.7E-04 |
| rs8079220 | 17 | 58316733 | *USP32* | C/T |  | 0.15 | 0.129 | 9.6E-01 | 2637 | 7.7E-04 |
| rs5744934 | 12 | 133220526 | *POLE* | T/C |  | 0.82 | -0.119 | 7.8E-01 | 2637 | 8.2E-04 |
| rs2020862 | 1 | 171168584 | *FMO2* | C/T |  | 0.48 | -0.092 | 7.3E-01 | 2637 | 8.4E-04 |
| rs61742644 | 10 | 50018765 | *WDFY4* | G/A |  | 0.05 | 0.204 | 2.5E-01 | 2637 | 8.5E-04 |
| rs2006996 | 9 | 117592638 | *TNFSF15* | T/C |  | 0.91 | -0.158 | 5.2E-01 | 2637 | 9.3E-04 |
| rs3806164 | 1 | 3380057 | *ARHGEF16* | G/A |  | 0.02 | -0.324 | 7.7E-01 | 2637 | 9.3E-04 |
| rs1369023 | 1 | 98993058 | *SNX7* | C/T |  | 0.66 | 0.121 | NA | 1660 | 9.5E-04 |
| rs111605439 | 6 | 35745305 | *CLPSL2* | G/A |  | 0.07 | 0.170 | 4.2E-01 | 2637 | 9.6E-04 |

**Abbreviations: Chr – chromosome; Pos – position; E/A – effect and alternative alleles; EAF – effect allele frequencies; β WHR – β coefficients per change of WHR-increasing allele on WHR (adjusted for BMI, inverse normal transformed ranked scale); Het P value – for heterogeneity in the meta-analysis; *n* – number of participants; P value – for association with WHR.**

**Supplementary Table 6B. South Asian exome-array analysis – Replication of top ranking single markers in South Asians (GWAS) and Europeans (GIANT consortium meta-analysis (20)).**

|  |  |  |  | **SOUTH ASIAN GWAS** | | | |  | **EUROPEANS REPORTED** | | | | | | |
| --- | --- | --- | --- | --- | --- | --- | --- | --- | --- | --- | --- | --- | --- | --- | --- |
| **MarkerName** | **Nearest Gene** | **E/A** |  | **EAF** | **β (SEM) WHR** | ***n*** | **P value** |  | **Lead SNP** | **R^2^** | **E/A** | **EAF** | **β (SEM) WHR** | ***n*** | **P value** |
|  |  |  |  |  |  |  |  |  |  |  |  |  |  |  |  |
|  |  |  |  |  |  |  |  |  |  |  |  |  |  |  |  |
| rs34996750 | *ANKRD12* | T/C |  | NA | NA | NA | NA |  | rs34996750 | NA | T/C | . | 0.003 (0.071) | 1205 | 9.6E-01 |
| rs59550885 | *TGM7* | C/A |  | NA | NA | NA | NA |  | rs59550885 | NA | C/A | NA | NA | NA | NA |
| rs2018601 | *PABPC4L* | T/C |  | 0.53 | -0.008 (0.014) | 7632 | 5.8E-01 |  | rs2018601 | NA | T/C | 0.46 | 0.004 (0.007) | 34600 | 5.9E-01 |
| rs2149547 | *SHB* | G/A |  | 0.40 | -0.008 (0.014) | 8070 | 5.6E-01 |  | rs2149547 | NA | G/A | 0.43 | -0.003 (0.007) | 34601 | 6.5E-01 |
| rs1236913 | *PTGS1* | T/C |  | 0.18 | 0.013 (0.018) | 7633 | 4.9E-01 |  | rs1236913 | NA | T/C | 0.07 | -0.018 (0.016) | 28058 | 2.4E-01 |
| rs142639450 | *COL9A3* | G/A |  | 0.05 | -0.002 (0.044) | 7633 | 9.7E-01 |  | rs142639450 | NA | G/A | NA | NA | NA | NA |
| rs8147 | *DNAJB11* | A/G |  | 0.75 | -0.023 (0.016) | 7633 | 1.5E-01 |  | rs8147 | NA | A/G | 0.83 | -0.003 (0.009) | 34595 | 7.6E-01 |
| rs10511089 | *CADM2* | G/T |  | 0.09 | 0.007 (0.023) | 8070 | 7.5E-01 |  | rs10511089 | NA | G/T | 0.10 | -0.01 (0.01) | 34599 | 3.3E-01 |
| rs12638212 | *MYH15* | G/A |  | 0.16 | -0.005 (0.018) | 8070 | 7.7E-01 |  | rs12638212 | NA | G/A | 0.05 | -0.007 (0.015) | 34602 | 6.4E-01 |
| rs34123504 | *BCL9L* | A/G |  | NA | NA | NA | NA |  | rs34123504 | NA | A/G | NA | NA | NA | NA |
| rs3102817 | *AUTS2* | C/A |  | 0.38 | 0.006 (0.014) | 8070 | 6.5E-01 |  | rs3102817 | NA | C/A | 0.43 | 0.002 (0.007) | 34590 | 7.4E-01 |
| rs498573 | *ETS1* | C/T |  | 0.73 | 0.015 (0.015) | 7633 | 3.4E-01 |  | rs498573 | NA | C/T | 0.83 | 0.003 (0.009) | 34643 | 7.3E-01 |
| rs34458979 | *SETBP1* | T/C |  | 0.70 | 0.016 (0.016) | 7633 | 3.4E-01 |  | rs34458979 | NA | T/C | NA | NA | NA | NA |
| rs12712508 | *CRIM1* | A/G |  | 0.72 | -0.031 (0.015) | 8069 | 4.3E-02 |  | rs12712508 | NA | A/G | 0.66 | 0.011 (0.007) | 34596 | 1.1E-01 |
| rs2228305 | *FASN* | C/T |  | 0.08 | -0.027 (0.031) | 8069 | 3.8E-01 |  | rs2228305 | NA | C/T | NA | NA | NA | NA |
| rs890947 | *CD180* | G/A |  | 0.58 | 0.012 (0.013) | 8070 | 3.8E-01 |  | rs890947 | NA | G/A | 0.53 | -0.004 (0.007) | 34601 | 5.9E-01 |
| rs200294583 | *FMNL3* | C/A |  | NA | NA | NA | NA |  | rs200294583 | NA | C/A | NA | NA | NA | NA |
| rs73038948 | *CAPN12* | T/C |  | 0.84 | -0.014 (0.018) | 8069 | 4.5E-01 |  | rs11553600 | 1 | A/G | . | 0.011 (0.051) | 958 | 8.3E-01 |
| rs139962539 | *MASP2* | G/A |  | NA | NA | NA | NA |  | rs139962539 | NA | G/A | NA | NA | NA | NA |
| rs17778003 | *ZFAT* | C/T |  | 0.06 | -0.075 (0.028) | 8070 | 7.2E-03 |  | rs17778003 | NA | C/T | 0.11 | -0.006 (0.012) | 34602 | 5.8E-01 |
| rs2273510 | *CCDC28A* | C/T |  | 0.02 | -0.035 (0.055) | 7633 | 5.3E-01 |  | rs2273510 | NA | C/T | 0.03 | -0.002 (0.026) | 30319 | 9.4E-01 |
| rs3085 | *CR1L* | A/G |  | 0.78 | 0.026 (0.017) | 7633 | 1.1E-01 |  | rs3085 | NA | A/G | . | 0.007 (0.011) | 34602 | 5.2E-01 |
| rs6794 | *EXOSC7* | G/C |  | 0.72 | -0.016 (0.015) | 8069 | 3.1E-01 |  | rs6794 | NA | G/C | 0.53 | 0.01 (0.007) | 34601 | 1.5E-01 |
| rs138658798 | *ITIH5* | C/T |  | 0.01 | -0.106 (0.073) | 7633 | 1.5E-01 |  | rs138658798 | NA | C/T | NA | NA | NA | NA |
| rs57971665 | *PTPRQ* | G/A |  | NA | NA | NA | NA |  | rs57971665 | NA | G/A | NA | NA | NA | NA |
| rs8079220 | *USP32* | C/T |  | 0.16 | 0.019 (0.019) | 8070 | 3.1E-01 |  | rs8079220 | NA | C/T | 0.21 | 0.003 (0.009) | 34574 | 7.0E-01 |
| rs5744934 | *POLE* | T/C |  | 0.84 | 0.006 (0.019) | 7633 | 7.7E-01 |  | rs5744934 | NA | T/C | 0.92 | 0.003 (0.012) | 34598 | 7.9E-01 |
| rs2020862 | *FMO2* | C/T |  | 0.46 | -0.005 (0.013) | 8070 | 6.9E-01 |  | rs2020862 | NA | C/T | . | 0.008 (0.007) | 34595 | 2.6E-01 |
| rs61742644 | *WDFY4* | G/A |  | 0.05 | -0.003 (0.033) | 7633 | 9.3E-01 |  | rs7068756 | 1 | A/G | 0.97 | 0.006 (0.01) | 74122 | 5.5E-01 |
| rs2006996 | *TNFSF15* | T/C |  | 0.92 | 0.014 (0.024) | 8070 | 5.8E-01 |  | rs2006996 | NA | T/C | 0.95 | 0.01 (0.015) | 32586 | 5.2E-01 |
| rs3806164 | *ARHGEF16* | G/A |  | 0.03 | -0.013 (0.041) | 8070 | 7.5E-01 |  | rs3806164 | NA | G/A | . | 0.009 (0.023) | 31865 | 7.0E-01 |
| rs1369023 | *SNX7* | C/T |  | 0.67 | 0.022 (0.015) | 7632 | 1.4E-01 |  | rs1369023 | NA | C/T | NA | NA | NA | NA |
| rs111605439 | *CLPSL2* | G/A |  | NA | NA | NA | NA |  | rs111605439 | NA | G/A | NA | NA | NA | NA |

**Abbreviations: Chr – chromosome; Pos – position; E/A – effect and alternative alleles; EAF – effect allele frequencies; β (SEM) – β coefficients (standard error of mean) per change of WHR-increasing allele on WHR (adjusted for BMI, inverse normal transformed ranked scale); Het P value – for heterogeneity in the meta-analysis; *n* – number of participants; P value – for association with WHR; Lead SNP – representative SNP at the discovery locus used in the replication analysis; R^2^ – linkage disequilibrium between lead and discovery SNP.**
