# Supplementary material for: Investigation of Genetic Variation Underlying Central Obesity amongst South Asians
Source: PLoS One. 2016 May 19;11(5):e0155478. doi: 10.1371/journal.pone.0155478 (PMC4873263; doi:10.1371/journal.pone.0155478)
Supplement: S7 Table — (DOCX) [file pone.0155478.s014.docx]

**Supplementary Table 7. South Asian exome-array analysis – Top ranking gene-based association results at P<1x10^-3^.**

| **Gene-based test** | **Gene Locus** | **Number of variants** | **Mean MAF** | **β WHR** | **P value** | **MAF cutoff** |
| --- | --- | --- | --- | --- | --- | --- |
|  |  |  |  |  |  |  |
| **CMC** | *SMOX* | 2 | 0.0002 | 2.950 | 3.0E-05 | n/a |
|  | *TARBP1* | 13 | 0.0025 | 0.298 | 1.5E-04 | n/a |
|  | *PAK3* | 1 | 0.0385 | -0.210 | 1.7E-04 | n/a |
|  | *TGM7* | 8 | 0.0068 | -0.178 | 2.3E-04 | n/a |
|  | *IARS2* | 3 | 0.0082 | 0.322 | 2.7E-04 | n/a |
|  | *CCDC28A* | 4 | 0.0072 | -0.289 | 3.8E-04 | n/a |
|  |  |  |  |  |  |  |
| **MB** | *SMOX* | 2 | 0.0002 | 0.041 | 3.0E-05 | n/a |
|  | *CCDC151* | 3 | 0.0152 | -0.029 | 3.8E-04 | n/a |
|  | *RSAD2* | 2 | 0.0005 | 0.034 | 4.2E-04 | n/a |
|  | *CNGB3* | 6 | 0.0125 | 0.020 | 5.6E-04 | n/a |
|  | *ANGPTL4* | 5 | 0.0028 | -0.021 | 6.2E-04 | n/a |
|  |  |  |  |  |  |  |
| **VT** | *SMOX* | 2 | 0.0002 | 2.950 | 5.8E-05 | 0.0002 |
|  | *CCDC151* | 2 | 0.0046 | -0.578 | 1.7E-04 | 0.0087 |
|  | *DNAH8* | 18 | 0.0061 | -0.175 | 7.9E-04 | 0.0292 |
|  | *IARS2* | 3 | 0.0082 | 0.322 | 8.4E-04 | 0.0231 |
|  |  |  |  |  |  |  |
| **SKAT** | *TGM7* | 8 | 0.0068 | 788205* | 7.4E-05 | n/a |
|  | *SMOX* | 2 | 0.0002 | 11065* | 1.4E-04 | n/a |
|  | *POLR1C* | 2 | 0.0007 | 40997* | 2.9E-04 | n/a |
|  | *RDH16* | 3 | 0.0004 | 32451* | 3.4E-04 | n/a |
|  | *ZNF598* | 12 | 0.0015 | 252530* | 3.9E-04 | n/a |

**Abbreviations: CMC – combined multivariate and collapsing; MB – Madsen-Browning; VT – variable threshold; SKAT – sequence kernel association test; Mean MAF – average minor allele frequencies; β WHR - β coefficients for gene-based effects on WHR (adjusted for BMI, inverse normal transformed ranked scale normalised), *SKAT test statistic; P value – for association with WHR; MAF cutoff – permutation-based MAF threshold.**
