# Supplementary material for: Investigation of Genetic Variation Underlying Central Obesity amongst South Asians
Source: PLoS One. 2016 May 19;11(5):e0155478. doi: 10.1371/journal.pone.0155478 (PMC4873263; doi:10.1371/journal.pone.0155478)
Supplement: S8 Table — (DOCX) [file pone.0155478.s015.docx]

**Table S8. Comparison of 48 known WHR SNPs in South Asians and Europeans, extended analysis (N=** **12,240).**

| **Marker Name** | **Nearest Gene** | **E/A** | **Ref** |  | **EAF** | **β (SEM)** | **P value** | ***n*** | **Dir** |  | **EAF** | **β (SEM)** | **P value** | ***n*** | **Dir** |  | **EAF** | **β (SEM)** | **P value** | ***n*** | **Dir** |  | **EAF** | **β (SEM)** | **P value** | ***n*** |
| --- | --- | --- | --- | --- | --- | --- | --- | --- | --- | --- | --- | --- | --- | --- | --- | --- | --- | --- | --- | --- | --- | --- | --- | --- | --- | --- |
|  |  |  |  |  |  |  |  |  |  |  |  |  |  |  |  |  |  |  |  |  |  |  |  |  |  |  |
|  |  |  |  |  | **SOUTH ASIANS DISCOVERY** | | | | |  | **SOUTH ASIANS REPLICATION** | | | | |  | **SOUTH ASIANS ALL** | | | | |  | **EUROPEAN REPORTED** | | | |
| **rs9491696** | *RSPO3* | G/C | (19) |  | 0.49 | -0.011 (0.012) | 3.5E-01 | 10,318 | - |  | 0.44 | 0.049 (0.038) | 2.0E-01 | 1,922 | + |  | 0.49 | -0.006 (0.011) | 6.0E-01 | 12,240 | - |  | 0.48 | 0.042 | 1.8E-40 | 113,582 |
| **rs6905288** | *VEGFA* | A/G | (19) |  | 0.81 | 0.014 (0.018) | 4.2E-01 | 10,318 | + |  | 0.80 | 0.006 (0.051) | 9.1E-01 | 1,922 | + |  | 0.81 | 0.013 (0.017) | 4.2E-01 | 12,240 | + |  | 0.56 | 0.036 | 5.9E-25 | 95,430 |
| **rs984222** | *TBX15-WARS2* | G/C | (19) |  | 0.48 | 0.032 (0.012) | 6.6E-03 | 10,318 | + |  | 0.44 | 0.029 (0.035) | 4.2E-01 | 1,922 | + |  | 0.47 | 0.032 (0.011) | 4.8E-03 | 12,240 | + |  | 0.64 | 0.034 | 8.7E-25 | 109,623 |
| **rs1055144** | *NFE2L3* | T/C | (19) |  | 0.39 | 0.018 (0.012) | 1.3E-01 | 10,318 | + |  | 0.38 | -0.048 (0.036) | 1.8E-01 | 1,922 | - |  | 0.39 | 0.011 (0.011) | 3.2E-01 | 12,240 | + |  | 0.21 | 0.04 | 1.0E-24 | 113,636 |
| **rs10195252** | *GRB14* | T/C | (19) |  | 0.72 | -0.005 (0.013) | 7.0E-01 | 10,318 | - |  | 0.69 | 0.004 (0.040) | 9.3E-01 | 1,922 | + |  | 0.72 | -0.004 (0.012) | 7.4E-01 | 12,240 | - |  | 0.6 | 0.033 | 2.1E-24 | 102,449 |
| **rs4846567** | *LYPLAL1* | G/T | (19) |  | 0.76 | 0.008 (0.014) | 5.6E-01 | 10,318 | + |  | 0.74 | -0.015 (0.047) | 7.5E-01 | 1,922 | - |  | 0.76 | 0.006 (0.013) | 6.4E-01 | 12,240 | + |  | 0.72 | 0.034 | 6.9E-21 | 91,820 |
| **rs1011731** | *DNM3-PIGC* | G/A | (19) |  | 0.44 | 0.028 (0.012) | 1.8E-02 | 10,318 | + |  | 0.44 | -0.031 (0.035) | 3.8E-01 | 1,922 | - |  | 0.44 | 0.022 (0.011) | 5.1E-02 | 12,240 | + |  | 0.43 | 0.028 | 9.5E-18 | 92,018 |
| **rs718314** | *ITPR2-SSPN* | G/A | (19) |  | 0.24 | 0.003 (0.014) | 8.1E-01 | 10,318 | + |  | 0.24 | 0.102 (0.043) | 1.7E-02 | 1,922 | + |  | 0.24 | 0.013 (0.013) | 3.3E-01 | 12,240 | + |  | 0.26 | 0.03 | 1.1E-17 | 107,503 |
| **rs1294421** | *LY86* | G/T | (19) |  | 0.45 | -0.007 (0.039) | 8.5E-01 | 10,318 | - |  | 0.23 | -0.142 (0.242) | 5.6E-01 | 394 | - |  | 0.44 | -0.011 (0.038) | 8.5E-01 | 10,712 | - |  | 0.61 | 0.028 | 1.8E-17 | 102,189 |
| **rs1443512** | *HOXC13* | A/C | (19) |  | 0.32 | 0.002 (0.013) | 9.0E-01 | 10,318 | + |  | 0.32 | 0.058 (0.038) | 1.3E-01 | 1,922 | + |  | 0.32 | 0.007 (0.012) | 5.4E-01 | 12,240 | + |  | 0.24 | 0.031 | 6.4E-17 | 112,353 |
| **rs4823006** | *ZNRF3-KREMEN1* | A/G | (19) |  | 0.44 | -0.009 (0.012) | 4.8E-01 | 10,318 | - |  | 0.37 | 0.043 (0.038) | 2.6E-01 | 1,922 | + |  | 0.43 | -0.004 (0.012) | 7.4E-01 | 12,240 | - |  | 0.57 | 0.023 | 1.1E-11 | 93,911 |
| **rs6784615** | *NISCH-STAB1* | T/C | (19) |  | 0.96 | -0.037 (0.03) | 2.1E-01 | 10,318 | - |  | 0.96 | 0.004 (0.091) | 9.7E-01 | 1,922 | + |  | 0.96 | -0.033 (0.028) | 2.4E-01 | 12,240 | - |  | 0.94 | 0.043 | 3.8E-10 | 109,028 |
| **rs6861681** | *CPEB4* | A/G | (19) |  | 0.13 | 0.007 (0.017) | 6.7E-01 | 10,318 | + |  | 0.14 | 0.011 (0.054) | 8.4E-01 | 1,922 | + |  | 0.13 | 0.008 (0.016) | 6.4E-01 | 12,240 | + |  | 0.34 | 0.022 | 1.9E-09 | 85,722 |
| **rs6795735** | *ADAMTS9* | C/T | (19) |  | 0.28 | -0.002 (0.014) | 8.6E-01 | 10,318 | - |  | 0.33 | -0.121 (0.038) | 1.4E-03 | 1,922 | - |  | 0.29 | -0.016 (0.013) | 2.2E-01 | 12,240 | - |  | 0.59 | 0.025 | 1.1E+13 | 84,480 |
| **rs4765219** | *CCDC92* | C/A | (22) |  | 0.79 | 0.005 (0.015) | 7.2E-01 | 10,005 | + |  | 0.77 | 0.001 (0.044) | 9.8E-01 | 1,922 | + |  | 0.78 | 0.005 (0.014) | 7.2E-01 | 11,927 | + |  | 0.668 | 0.028 (0.004) | 1.6E-15 | 209,807 |
| **rs979012** | *BMP2* | T/C | (22) |  | 0.44 | 0.017 (0.012) | 1.7E-01 | 10,005 | + |  | 0.42 | 0.035 (0.035) | 3.1E-01 | 1,922 | + |  | 0.44 | 0.019 (0.012) | 1.0E-01 | 11,927 | + |  | 0.3425 | 0.027 (0.004) | 3.3E-14 | 209,941 |
| **rs17451107** | *LEKR1* | T/C | (22) |  | 0.73 | 0.010 (0.014) | 4.9E-01 | 9,504 | + |  | 0.70 | 0.027 (0.040) | 5.0E-01 | 1,922 | + |  | 0.73 | 0.012 (0.013) | 3.9E-01 | 11,426 | + |  | 0.6145 | 0.026 (0.004) | 1.1E-12 | 207,795 |
| **rs4081724** | *CEBPA* | G/A | (22) |  | 0.96 | 0.031 (0.031) | 3.1E-01 | 9,505 | + |  | 0.91 | -0.030 (0.084) | 7.2E-01 | 1,922 | - |  | 0.95 | 0.024 (0.029) | 4.1E-01 | 11,427 | + |  | 0.8512 | 0.035 (0.005) | 7.4E-12 | 207,418 |
| **rs4646404** | *PEMT* | G/A | (22) |  | 0.87 | -0.031 (0.019) | 1.0E-01 | 9505 | - |  | 0.84 | -0.073 (0.051) | 1.5E-01 | 1,922 | - |  | 0.87 | -0.036 (0.018) | 4.1E-02 | 11,427 | - |  | 0.6653 | 0.027 (0.004) | 1.4E-11 | 198,196 |
| **rs12679556** | *MSC* | G/T | (22) |  | 0.44 | 0.021 (0.012) | 9.0E-02 | 10,005 | + |  | 0.42 | -0.015 (0.036) | 6.8E-01 | 1,922 | - |  | 0.43 | 0.017 (0.011) | 1.4E-01 | 11,927 | + |  | 0.2475 | 0.027 (0.004) | 2.1E-11 | 203,826 |
| **rs7759742** | *BTNL2* | A/T | (22) |  | 0.58 | -0.005 (0.013) | 7.0E-01 | 9,504 | - |  | 0.54 | 0.029 (0.038) | 4.4E-01 | 1,528 | + |  | 0.57 | -0.001 (0.012) | 9.0E-01 | 11,032 | - |  | 0.511 | 0.023 (0.003) | 4.4E-11 | 208,263 |
| **rs6090583** | *EYA2* | A/G | (22) |  | 0.54 | -0.015 (0.013) | 2.5E-01 | 9,505 | - |  | 0.53 | 0.013 (0.035) | 7.1E-01 | 1,922 | + |  | 0.54 | -0.012 (0.012) | 3.5E-01 | 11,427 | - |  | 0.48 | 0.022 (0.003) | 6.2E-11 | 209,435 |
| **rs1440372** | *SMAD6* | C/T | (22) |  | 0.74 | 0.009 (0.014) | 5.3E-01 | 10,005 | + |  | 0.75 | -0.023 (0.041) | 5.7E-01 | 1,922 | - |  | 0.74 | 0.005 (0.013) | 6.8E-01 | 11,927 | + |  | 0.7097 | 0.024 (0.004) | 1.1E-10 | 207,447 |
| **rs7801581** | *HOXA11* | T/C | (22) |  | NA | NA | NA | NA | NA |  | NA | NA | NA | NA | NA |  | NA | NA | NA | NA | NA |  | 0.2424 | 0.027 (0.004) | 3.7E-10 | 195,215 |
| **rs1569135** | *CALCRL* | A/G | (22) |  | 0.43 | 0.010 (0.012) | 4.0E-01 | 10,005 | + |  | 0.43 | -0.015 (0.035) | 6.8E-01 | 1,922 | - |  | 0.43 | 0.008 (0.012) | 5.1E-01 | 11,927 | + |  | 0.5297 | 0.021 (0.003) | 5.6E-10 | 209,906 |
| **rs905938** | *DCST2* | T/C | (22) |  | 0.85 | 0.003 (0.017) | 8.6E-01 | 9,505 | + |  | 0.81 | -0.021 (0.049) | 6.6E-01 | 1,922 | - |  | 0.84 | 0.000 (0.016) | 9.9E-01 | 11,427 | + |  | 0.7427 | 0.025 (0.004) | 7.3E-10 | 207,867 |
| **rs12608504** | *JUND* | A/G | (22) |  | 0.33 | 0.028 (0.013) | 2.7E-02 | 10,005 | + |  | 0.35 | -0.044 (0.037) | 2.3E-01 | 1,922 | - |  | 0.34 | 0.021 (0.012) | 8.7E-02 | 11,927 | + |  | 0.355 | 0.022 (0.004) | 8.8E-10 | 209,990 |
| **rs8042543** | *KLF13* | C/T | (22) |  | 0.79 | 0.017 (0.016) | 2.7E-01 | 9,505 | + |  | 0.77 | 0.098 (0.044) | 2.6E-02 | 1,922 | + |  | 0.78 | 0.026 (0.015) | 7.5E-02 | 11,427 | + |  | 0.7824 | 0.026 (0.004) | 1.2E-09 | 208,255 |
| **rs1385167** | *MEIS1* | G/A | (22) |  | 0.18 | 0.019 (0.016) | 2.2E-01 | 10,005 | + |  | 0.21 | 0.049 (0.048) | 3.0E-01 | 1,922 | + |  | 0.18 | 0.022 (0.015) | 1.4E-01 | 11,927 | + |  | 0.1475 | 0.029 (0.005) | 1.9E-09 | 206,619 |
| **rs10919388** | *GORAB* | C/A | (22) |  | 0.75 | 0.003 (0.014) | 8.3E-01 | 9,505 | + |  | 0.73 | 0.033 (0.041) | 4.1E-01 | 1,922 | + |  | 0.74 | 0.006 (0.013) | 6.4E-01 | 11,427 | + |  | 0.7226 | 0.024 (0.004) | 3.2E-09 | 181,049 |
| **rs10804591** | *PLXND1* | A/C | (22) |  | 0.65 | 0.005 (0.013) | 6.7E-01 | 10,005 | + |  | 0.66 | -0.007 (0.037) | 8.5E-01 | 1,922 | - |  | 0.65 | 0.004 (0.012) | 7.3E-01 | 11,927 | + |  | 0.795 | 0.025 (0.004) | 6.6E-09 | 209,921 |
| **rs8030605** | *RFX7* | A/G | (22) |  | 0.26 | -0.007 (0.014) | 6.0E-01 | 10,005 | - |  | 0.27 | -0.010 (0.042) | 8.2E-01 | 1,922 | - |  | 0.26 | -0.008 (0.013) | 5.7E-01 | 11,927 | - |  | 0.1415 | 0.030 (0.005) | 8.8E-09 | 208,374 |
| **rs10991437** | *ABCA1* | A/C | (22) |  | 0.09 | 0.003 (0.021) | 8.9E-01 | 10,005 | + |  | 0.18 | 0.040 (0.065) | 5.4E-01 | 1,922 | + |  | 0.11 | 0.007 (0.020) | 7.5E-01 | 11,927 | + |  | 0.1135 | 0.031 (0.005) | 1.0E-08 | 209,941 |
| **rs224333** | *GDF5* | G/A | (22) |  | 0.44 | 0.012 (0.013) | 3.2E-01 | 10,004 | + |  | 0.47 | -0.037 (0.039) | 3.4E-01 | 1,922 | - |  | 0.45 | 0.008 (0.012) | 5.2E-01 | 11,926 | + |  | 0.6232 | 0.020 (0.004) | 2.6E-08 | 208,025 |
| **rs6556301** | *FGFR4* | T/G | (22) |  | 0.41 | 0.034 (0.013) | 6.3E-03 | 9,505 | + |  | 0.42 | -0.020 (0.036) | 5.7E-01 | 1,922 | - |  | 0.41 | 0.028 (0.012) | 1.7E-02 | 11,427 | + |  | 0.3573 | 0.022 (0.004) | 2.6E-08 | 178,874 |
| **rs303084** | *SPATA5-FGF2* | A/G | (22) |  | 0.73 | 0.019 (0.014) | 1.6E-01 | 10,005 | + |  | 0.69 | 0.005 (0.039) | 9.0E-01 | 1,922 | + |  | 0.73 | 0.017 (0.013) | 1.7E-01 | 11,927 | + |  | 0.7961 | 0.023 (0.004) | 3.9E-08 | 209,941 |
| **rs9991328** | *FAM13A* | T/C | (22) |  | 0.58 | 0.002 (0.012) | 8.7E-01 | 10,005 | + |  | 0.57 | -0.028 (0.036) | 4.4E-01 | 1,922 | - |  | 0.58 | -0.001 (0.011) | 9.3E-01 | 11,927 | - |  | 0.4882 | 0.019 (0.003) | 4.5E-08 | 209,925 |
| **rs11231693** | *MACROD1-VEGFB* | A/G | (22) |  | 0.03 | 0.041 (0.042) | 3.2E-01 | 9,505 | + |  | 0.12 | -0.068 (0.128) | 5.9E-01 | 1,922 | - |  | 0.04 | 0.031 (0.040) | 4.4E-01 | 11,427 | + |  | 0.0624 | 0.041 (0.008) | 4.5E-08 | 198,072 |
|  |  |  |  |  |  |  |  |  | **27/37** |  |  |  |  |  | **19/37** |  |  |  |  |  | **26/37** |  |  |  |  |  |
|  |  |  |  |  |  | | | | |  |  | | | | |  |  | | | | |  |  | | | |
|  |  |  |  |  | **SOUTH ASIANS WOMEN LOLIPOP** | | | | |  | **SOUTH ASIANS WOMEN REPLICATION** | | | | |  | **SOUTH ASIANS WOMEN ALL** | | | | |  | **EUROPEAN WOMEN REPORTED** | | | |
| **rs4684854** | *PPARG* | C/G | (20) |  | 0.61 | 0.088 (0.035) | 1.3E-02 | 1,463 | + |  | 0.58 | -0.049 (0.055) | 3.7E-01 | 900 | - |  | 0.60 | 0.048 (0.029) | 1.0E-01 | 2,363 | + |  | 0.43 | 0.037 | 4.2E-14 | 96,472 |
| **rs10478424** | *HSD17B4* | A/T | (20) |  | 0.73 | -0.026 (0.039) | 5.1E-01 | 1,463 | - |  | 0.68 | -0.071 (0.057) | 2.2E-01 | 900 | - |  | 0.71 | -0.040 (0.032) | 2.1E-01 | 2,363 | - |  | 0.78 | 0.039 | 3.5E-09 | 73,066 |
| **rs7830933** | *NKX2-6* | A/G | (22) |  | 0.73 | 0.073 (0.041) | 7.3E-02 | 1,443 | + |  | 0.73 | 0.062 (0.061) | 3.1E-01 | 900 | + |  | 0.73 | 0.070 (0.034) | 4.1E-02 | 2,343 | + |  | 0.77 | 0.037 (0.005) | 1.2E-12 | 116,567 |
| **rs9687846** | *MAP3K1* | A/G | (22) |  | 0.17 | -0.018 (0.047) | 7.1E-01 | 1,443 | - |  | 0.20 | 0.025 (0.073) | 7.3E-01 | 900 | + |  | 0.18 | -0.005 (0.040) | 8.9E-01 | 2,343 | - |  | 0.19 | 0.041 (0.006) | 3.8E-12 | 115,897 |
| **rs2925979** | *CMIP* | T/C | (22) |  | 0.30 | 0.051 (0.038) | 1.7E-01 | 1,443 | + |  | 0.33 | 0.076 (0.054) | 1.6E-01 | 900 | + |  | 0.31 | 0.059 (0.031) | 5.6E-02 | 2,343 | + |  | 0.31 | 0.032 (0.005) | 3.4E-11 | 115,431 |
| **rs12454712** | *BCL2* | T/C | (22) |  | 0.45 | 0.043 (0.034) | 2.1E-01 | 1,443 | + |  | 0.41 | 0.068 (0.051) | 1.8E-01 | 900 | + |  | 0.43 | 0.051 (0.028) | 7.3E-02 | 2,343 | + |  | 0.61 | 0.035 (0.006) | 1.1E-09 | 96,182 |
| **rs8066985** | *KCNJ2* | A/G | (22) |  | 0.43 | 0.006 (0.034) | 8.7E-01 | 1,443 | + |  | 0.43 | -0.024 (0.055) | 6.7E-01 | 900 | - |  | 0.43 | -0.002 (0.029) | 9.4E-01 | 2,343 | - |  | 0.50 | 0.026 (0.005) | 4.0E-09 | 116,683 |
| **rs7917772** | *SFXN2* | A/G | (22) |  | 0.40 | 0.031 (0.036) | 3.8E-01 | 1,443 | + |  | 0.37 | -0.062 (0.053) | 2.5E-01 | 900 | - |  | 0.39 | 0.002 (0.030) | 9.5E-01 | 2,343 | + |  | 0.62 | 0.027 (0.005) | 5.5E-09 | 116,514 |
| **rs1776897** | *HMGA1* | G/T | (22) |  | 0.05 | 0.055 (0.077) | 4.7E-01 | 1,443 | + |  | 0.11 | 0.160 (0.098) | 1.0E-01 | 900 | + |  | 0.07 | 0.095 (0.061) | 1.2E-01 | 2,343 | + |  | 0.08 | 0.052 (0.009) | 6.8E-09 | 100,516 |
| **rs3805389** | *NMU* | A/G | (22) |  | 0.26 | -0.019 (0.039) | 6.3E-01 | 1,443 | - |  | 0.26 | 0.106 (0.057) | 6.1E-02 | 900 | + |  | 0.26 | 0.021 (0.032) | 5.1E-01 | 2,343 | + |  | 0.28 | 0.027 (0.005) | 4.6E-08 | 116,226 |
| **rs1534696** | *SNX10* | C/A | (22) |  | 0.42 | -0.087 (0.039) | 2.7E-02 | 1,443 | - |  | 0.41 | 0.001 (0.061) | 9.8E-01 | 900 | + |  | 0.41 | -0.061 (0.033) | 6.2E-02 | 2,343 | - |  | 0.44 | 0.027 (0.005) | 5.4E-08 | 111,643 |
|  |  |  |  |  |  |  |  |  | **7/11** |  |  |  |  |  | **7/11** |  |  |  |  |  | **7/11** |  |  |  |  |  |
|  |  |  |  |  |  |  |  |  |  |  |  |  |  |  |  |  |  |  |  |  |  |  |  |  |  |  |

**Discovery – LOLIPOP Study; Replication – Sikh Diabetes Study (25) and Mauritius Study (28) combined; Reported – Europeans, GIANT consortium meta-analysis data (20).**

**Abbreviations: E/A – effect and alternative alleles; EAF - effect allele frequencies; β (SEM) - β coefficients (standard error of mean) per change of WHR-increasing allele on WHR (adjusted for BMI, inverse normal transformed ranked scale); P value - for association with WHR; Dir - direction of effect allele compared to reported European results.**
